# Barcode Sequencing Screen Identifies *SUB1* as a Regulator of Yeast Pheromone Inducible Genes

**DOI:** 10.1534/g3.115.026757

**Published:** 2016-02-01

**Authors:** Anna Sliva, Zheng Kuang, Pamela B. Meluh, Jef D. Boeke

**Affiliations:** * Institute for Systems Genetics, New York University Langone School of Medicine, New York 10016; † Human Genetics Program, Institute of Genetic Medicine, Johns Hopkins University School of Medicine, Baltimore, Maryland 21205; ‡ Calico Life Sciences, Google Inc., San Francisco, California 94080

**Keywords:** *SUB1*, mating, transcription

## Abstract

The yeast pheromone response pathway serves as a valuable model of eukaryotic mitogen-activated protein kinase (MAPK) pathways, and transcription of their downstream targets. Here, we describe application of a screening method combining two technologies: fluorescence-activated cell sorting (FACS), and barcode analysis by sequencing (Bar-Seq). Using this screening method, and p*FUS1*-GFP as a reporter for MAPK pathway activation, we readily identified mutants in known mating pathway components. In this study, we also include a comprehensive analysis of the *FUS1* induction properties of known mating pathway mutants by flow cytometry, featuring single cell analysis of each mutant population. We also characterized a new source of false positives resulting from the design of this screen. Additionally, we identified a deletion mutant, *sub1*Δ, with increased basal expression of p*FUS1*-GFP. Here, in the first ChIP-Seq of Sub1, our data shows that Sub1 binds to the promoters of about half the genes in the genome (tripling the 991 loci previously reported), including the promoters of several pheromone-inducible genes, some of which show an increase upon pheromone induction. Here, we also present the first RNA-Seq of a *sub1*Δ mutant; the majority of genes have no change in RNA, but, of the small subset that do, most show decreased expression, consistent with biochemical studies implicating Sub1 as a positive transcriptional regulator. The RNA-Seq data also show that certain pheromone-inducible genes are induced less in the *sub1*Δ mutant relative to the wild type, supporting a role for Sub1 in regulation of mating pathway genes. The *sub1*Δ mutant has increased basal levels of a small subset of other genes besides *FUS1*, including *IMD2* and *FIG1*, a gene encoding an integral membrane protein necessary for efficient mating.

The mating pathway in *Saccharomyces cerevisiae* signals through the well-studied mitogen-activated protein kinase (MAPK) pathway (Campbell *et al.* 2013). The functions of many genes within the pathway are known and characterized, and, notably, many of the MAPK pathway components were first identified in yeast (Bardwell 2004). Since its discovery, this pathway has served as a valuable model for the study of MAPK cascades. Importantly, many components of the yeast mating pathway, and factors necessary for efficient mating, have homologs in humans (Widmann *et al.* 1999), and our understanding of the process of yeast mating has led, and will continue to lead, to many beneficial applications. Further, the mating pathway is also a great system for studying regulated transcription. It is likely that not all the players have been identified, particularly those with only modest effects on expression of pheromone-inducible genes.

When a haploid yeast cell is stimulated by a mating pheromone of the opposite mating type, the receptor on the cell surface activates the pheromone MAPK signaling cascade. Ste12 is the major actor in stimulating the dramatic transcriptional change that results from the induction with mating pheromone, whereupon expression of about 200 genes is induced, and another 200 genes are repressed (Roberts *et al.* 2000). Many of the genes that increase expression under pheromone induction are genes needed for efficient mating, while genes repressed under induction with pheromone are enriched for genes that promote cell cycle progression, DNA replication, budding, and mitosis (Roberts *et al.* 2000).

Although many genes affecting the yeast mating pathway are known, it is likely that additional factors, which may or may not be pathway-specific, function to fine-tune and/or insulate the yeast MAPK pathway (Bardwell *et al.* 2007). To search for such factors that may have been missed by previous screening methods (Erdman *et al.* 1998; Chasse *et al.* 2006), we devised a multi-faceted screen to survey the mating pathway from a new perspective, similar to previous fluorescence-activated cell sorting (FACS)-based screens in yeast analyzed by deep sequencing of complex libraries (Sharon *et al.* 2012, 2014; Mogno *et al.* 2013). Specifically, we combined the use of an engineered MAP kinase pathway-responsive reporter (p*FUS1*-GFP) with FACS to screen a comprehensive library of yeast knockout (YKO) mutants. We then used Barcode analysis by sequencing (Bar-Seq) (Smith *et al.* 2009) to determine the relative representation of different mutants in each of several FACS-sorted pools prepared following induction with mating pheromone. In this way, we were able to identify mutants with altered patterns of reporter expression, and to show that it is possible to genetically analyze the dynamic process of response to mating pheromone. Further, our assay method utilizes the more sensitive and single-cell precision capabilities of flow cytometry, allowing us to identify deletion mutants with more subtle mutant *FUS1*-GFP phenotypes than the bulk *lacZ* assays used previously Chasse *et al.* 2006; Parrish *et al.* 2002; Bardwell *et al.* 1998; Posas and Saito 1997. This method is a valuable tool for identifying mutants with more subtle phenotypes that function in pathways to fine-tune gene expression.

Multiple screens were performed on the YKO library under both pheromone-exposed and vegetative growth conditions, with gating to select for outliers. While some of these screens efficiently recovered the vast majority of known genes in the mating pathway, others gave high rates of false positives. Here, we characterize an unusual source of false positives arising as a consequence of the specific design of the screen, perform an extensive FACS-based study of a wide variety of known individual mutants affecting the pathway, and identify a new mutant, *sub1*Δ, in which the basal transcription of the p*FUS1*-GFP reporter, as well as the RNA expression levels of another pheromone responsive gene, *FIG1*, is affected. We also perform the first ChIP-Seq of Sub1, show that it binds to the promoter regions of about half of the genes in the genome under vegetative growth conditions, and show that Sub1 is specifically recruited to the promoter region of *FUS1* and other pheromone-responsive genes upon induction with α-factor. We also perform RNA-Seq of a *sub1*Δ mutant and identify genes upregulated and downregulated compared to wild type. Finally, we show that, overall, the pheromone-inducible genes in the *sub1*Δ mutant are induced less under α-factor induction than wild type, further supporting a role for Sub1 in the regulation of pheromone-inducible genes.

## Materials and Methods

### Strains

The strains used in this study are listed in Supplemental Material, Table S7.

### Reporter design

A reporter with the promoter of *FUS1* driving expression of GFP (p*FUS1*-GFP) was assembled using the Gibson assembly method (Gibson *et al.* 2009) (Figure 2A). The reporter also contains a p*FUS1*-RFP gene that was not employed extensively in this study. The reporter has 1300 bp of homology flanking the *BAR1* locus, and, upon integration into the targeted locus, knocks out wild-type function of the Bar1 aspartyl protease. Since the wild-type function of Bar1 is to cleave α-factor, the knocking out of this gene allows for lower levels of α-factor to be used to induce the mating response, and allows for a sustained response. This design is ideal for this screen, as it allows for the simultaneous introduction of the reporter and knockout of *BAR1* in a single yeast transformation.

### Preparation of library

The reporter plasmid was linearized by digestion with *Bam*I-HF, and a high-efficiency transformation protocol (Pan *et al.* 2007) was used to transform the *MAT***a** haploid YKO library (Ooi *et al.* 2001). Transformants were selected on SC–Leu plates. After transformation of the YKO library with the reporter construct, and directly collecting/pooling the transformants, there was still a high level of background of Leu^–^ colonies. Therefore, we developed a protocol in which the transformants were replica-plated on SC–Leu plates, then scraped and aliquoted into –80° glycerol stocks (resulting in a YKO library with a final Leu^–^ background of 0.38%). Single colonies from the transformed YKO library were tested for correct integration at the *BAR1* locus using a Bar assay (Guthrie and Fink 1991), and the rate of integration at the correct locus was estimated at 97% (determined from 100 total colonies screened for Bar1 function).

### Library growth and induction with alpha-factor

An aliquot of the transformed YKO library was thawed, and an overnight timer was set to knock the cells into 200 ml SC–Leu medium (at a starting concentration of OD ∼0.1) to start growth at the appropriate time. The cells were grown to midlog phase in two separate flasks: one was induced with 10 nM α-factor for 2 hr, and the other was further grown vegetatively without addition of α-factor (Figure 2C). Three controls were likewise grown simultaneously with the library: untransformed BY4741 as the negative control, yAS38 (BY4741 transformed with the p*FUS1*-GFP reporter construct) as the basal control, and yAS38 induced with α-factor as the wild-type induced control. A 10-ml aliquot of cells was spun down at 3600 rpm for 2 min, washed twice with 10 ml of 1X PBS, resuspended in 1X PBS to a final concentration of ∼1 OD, and taken to the Bloomberg Johns Hopkins School of Public Health sorting facility, where cells were sorted on a Beckman Coulter MoFlo Cell sorter. An amount of ∼11 × 10e6 total starting cells was sorted into different populations based on GFP fluorescence (gating of populations used is shown in Figure S4), using the control strains as references. The sorted cells were then grown to saturation in 15 ml SC–Leu, spun down, and stored as glycerol stocks at –80°; ∼25 ODs of cells were used for gDNA extraction. The cells were first thawed at room temperature, and resuspended in 250 µl of reconstitution buffer (1 ml of resuspension solution concentrate, 0.18 g sorbitol, and 1 µl beta-mercaptoethanol). Reconstituted lyticase enzyme [1000 units (U)] was added to each tube, and each tube was mixed by inversion a few times. The tubes were incubated at room temperature for 1 hr with gentle mixing by inversion every 15 min. To isolate the gDNA, the Norgen Biotek Yeast Genomic DNA Isolation Kit (Product #18600) was used.

### Barcode sequencing

The UPTAG and DNTAG barcodes from the gDNAs were then PCR-amplified and sequenced to identify mutants enriched in the different populations. The primers that were used to amplify the UPTAG and DNTAG sequences (*Supplemental Methods*) are based on previous barcode sequencing studies (Smith *et al.* 2009). The primers include the TruSeq universal adaptors (allowing for the cluster formation for sequencing on the Illumina platform), a unique multiplex tag (allowing for pooling up to 18 samples in a single lane: nine UPTAG and nine DNTAG), and universal priming sites (used to amplify each barcode). Each PCR reaction was performed in a final volume of 100 μl, containing 1 U of Phusion polymerase, HF phusion buffer diluted to 1X, 250 µM dNTP, 250 nM of each primer, and ∼100 ng genomic DNA. The PCR amplification protocol used was: 30 sec at 98°; 25 cycles of 10 sec at 98°, 30 sec at 55°, 30 sec at 72°; 7 min at 72°; ∞ at 4°. A negative control was run for each PCR experiment to assure the absence of genomic DNA contamination. Additionally, in order to further take measures to avoid possible contamination of the PCR with genomic DNA, all PCR reactions were set up in the hood of a separate, clean room (with separate pipettes and reagents), where no yeast work was ever done. Lastly, the primers with the multiplex tag were rotated and assigned to different sorted populations for the different biological replicates to further reduce the possibility of data skewing due to use of a particular primer. The PCR reactions were run on a 2% agarose gel, gel extracted using the Zymoclean Gel DNA Recovery Kit, pooled in equimolar amounts, and sent for single-end, 50-cycle sequencing on an Illumina HiSeq. Three biological replicates of this experiment were performed for the “Un Gfp^–^” population (Table S1), the “In Gfp^–^” population (Table S2), the “In Gfp^basal^” (Table S3), and the “Un Gfp^+^” (Table S4) sorted populations. One sample was evaluated for the “In Gfp^++^” (Table S5) population. (This nomenclature is described in detail in Figure 2B and Figure 2C, and in the section *Ranking classes of knockout mutants* in *Results*).

### Barcode sequence analysis

For data analysis, we first clustered our reads from the sequencing run into the different sorted pools by demultiplexing the sequencing data. Next, we combined the barcodes listed on the yeast deletion website (YKOv2s: http://www-sequence.stanford.edu/group/yeast_deletion_project/YKOv2_info.txt) with the resequenced list (Smith *et al.* 2009), and used this combined list as a reference to identify which genes were present in each sorted population. We concatenated all barcodes together with 20 bp of ambiguous N bases between barcodes to form a reference, and aligned the reads to this sequence using Bowtie2 (Langmead and Salzberg 2012). We developed a program in R to count the number of reads for each barcode. We used 50 (UPTAG and/or DNTAG) reads in the control presorted YKO library as a minimum cutoff for including a specific deletion mutant in our data analysis. Using the DESeq package (Anders and Huber 2010), we generated a p-value for each gene, based on the number of reads from the sequencing run.

### Evaluation of individual mutants

To evaluate each individual mutant for p*FUS1*-GFP expression, a single colony was inoculated in 5 ml SC medium, and grown overnight. The following morning, the strain was diluted back to OD ∼0.1 in 5 ml SC medium, grown to midlog phase, and induced with 10 nM α-factor, or continued vegetative growth for 2 hr. The cells were then spun down at 3600 rpm, washed twice with 1X PBS, and GFP fluorescence was analyzed by flow cytometry.

### ChIP-Seq

For the ChIP-Seq experiments, wild type diploid BY4743 was transformed with the *bar1*Δ::p*FUS1*-GFP reporter, and a C-terminal 3HA tag targeting the native *SUB1* locus, similar to previous reports (Rosonina *et al.* 2009). This strain was sporulated, and a spore containing both the reporter and the 3HA tag, yAS420, was selected. We tested this strain for *SUB1* function by plating on moderate levels of sorbitol, and showed, by construction of double mutants (Rosonina *et al.* 2009), that the tagged strain retained some *SUB1* function (Figure S5). The strains were grown in 200 ml SC medium to midlog phase, and then treated with α-factor, 1M sorbitol, or untreated. The α-factor-treated cells were treated with 10 nM α-factor for 30 min, and the cells treated with sorbitol were spun down at 1000 g for 3 min, and resuspended in SC medium containing 1 M sorbitol for 5 min prior to crosslinking. Five samples were sequenced from the ChIP experiment: α-factor treated, no α-factor control, sorbitol, no sorbitol control, and an untagged strain (yAS38) as a negative control. We followed a ChIP protocol as previously described (Kuang *et al.* 2014); ∼50 OD cells were used for ChIP-Sequation, 5 µl of 0.4 mg/ml anti-HA 12CA5 (Roche) was used for the ChIP of Sub1-3HA. The KAPA Hyper Prep Kit for Illumina platforms was used for constructing the sequencing library. The ChIP-Seq samples were then sent for sequencing on the Illumina HiSeq for 50 cycles, single read. Reads were mapped using Bowtie2 against the SacCer2 reference sequence (UCSC Genome browser). CisGenome (Jiang *et al.* 2010) was used to visualize the ChIP-Seq data.

### RNA-Seq

To generate the strains used in RNA-Seq, BY4743 was transformed with the *bar1*Δ::p*FUS1*-GFP reporter, and *sub1*Δ was knocked out with the *kanMX* cassette. By sporulation, two isogenic *MAT***a** strains with the reporter were isolated, one with *sub1*Δ, yAS395, and a wild-type control (yAS418), and were used for performing RNA-Seq. For the RNA-Seq experiments, 150 ml cells were grown at 30° in SC medium to midlog phase. The α-factor-treated cells were induced with a total concentration of 10 nM α-factor for 45 min, or the strain was allowed to continue vegetative growth (uninduced control). For the high osmolarity response, the cells were grown to midlog phase, and spun down at 3000 rpm for 3 min, resuspended in SC medium containing 1 M sorbitol, or fresh SC medium (no sorbitol control), and growth was continued for 45 min; ∼5 OD cells were used for RNA-Seq. The QIAGEN RNeasy Mini Kit “purification of total RNA from yeast” protocol was used to isolate RNA from these samples. The RNA samples were then polyA-enriched, and sequenced using the Illumina HiSeq for 50 bp single reads. Reads were mapped using Bowtie2 against SacCer2. The number of reads that overlap genes was calculated in R, and the differential expression was analyzed by DESeq (Anders and Huber 2010).

### Data availability

The sequencing data from this manuscript is available under GEO accession number GSE71813.

## Results

### Evaluation of reporter and ranking of knockout mutants

**Figure 1:**
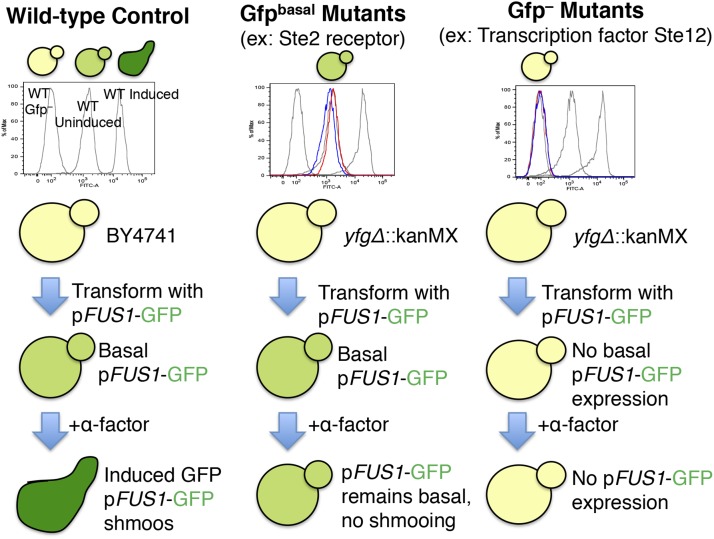
Overview of expected pheromone response pathway mutants. Control strains (gray), mutant uninduced (blue), and mutant induced with α-factor (red). The three wild-type controls were run with every experiment, in gray: wild-type Gfp^–^, Uninduced (“Un Gfp^basal^”), and Induced (“In Gfp^+^”).

Yeast strain BY4741, transformed with a reporter cassette (yAS38) expressing *FUS1*-GFP, serves as the wild-type control for these experiments. Wild-type yeast cells with an intact mating pathway express low but detectable basal levels of p*FUS1*-GFP (Gfp^basal^), and exhibit a significant increase in GFP expression upon induction with α-factor (to Gfp^+^ levels), as expected (Figure 1). *FUS1* is a haploid-specific gene; it is repressed in diploid cells and in mutants defective in silencing the silent mating loci *HML* and *HMR* (McCaffrey *et al.* 1987). Under the fluorescence microscope, the control reporter strain has the correct phenotype; upon treatment with α-factor, this strain changes morphology to form shmoos, and expresses higher levels of p*FUS1*-GFP (Figure S1). For assaying the *FUS1* expression profiles of mutants throughout this study, three control strains were run for each experiment: BY4741, a wild-type strain lacking the *FUS1* reporter grown under vegetative conditions (“Un Gfp^–^” control); BY4741 transformed with the reporter construct (yAS38) grown under vegetative condition expressing basal levels of *FUS1*-GFP (“Un Gfp^basal^” wild-type control); and the wild-type reporter strain treated with α-factor (“In Gfp^+^” wild-type control) (Figure 1). These control strains appear as three gray peaks in the histograms throughout this study.

### Ranking classes of knockout mutants

**Figure 2:**
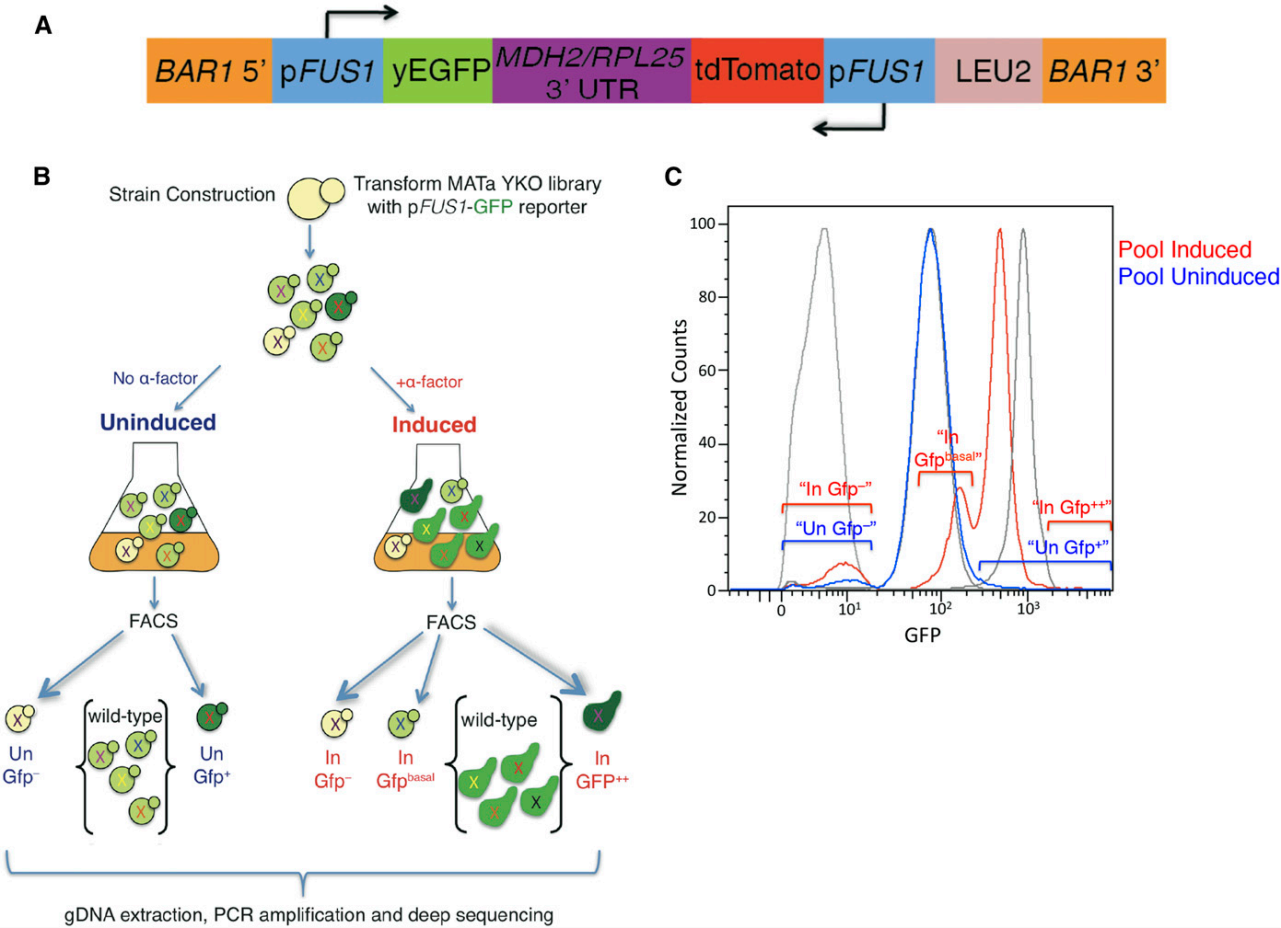
Overview of the screening strategy. (A) Reporter construct. (B) Overview of FACS sorting transformed YKO library. (C) FACS profile of YKO library overlay of no α-factor (blue), and the sorted mutant populations “Un Gfp^–^” and “Un Gfp^+^”. (D) FACS profile of YKO library overlay of +α-factor (red), and the sorted populations “in Gfp^–^”, “In Gfp^basal^”, and “In Gfp^++^” over wild-type controls (gray).

The GFP fluorescence profile of the YKO library transformed with the p*FUS1*-GFP reporter differs from that observed for the yAS38 control strain, suggesting that expression of *FUS1* is subject to positive and negative regulation under both uninduced and α-factor induced conditions. We expected and observed a number of distinct populations of cells in our evaluations of both uninduced and α-factor-induced cultures of cells bearing the reporter. The YKO library transformed with the reporter construct was either grown under vegetative conditions (Uninduced, “Un”), or treated with 10 nM α-factor for 2 hr (Induced, “In”). The library was then sorted based on different populations of cells with a mutant fluorescence profile. Ultimately, we chose to sort the mutants into five pools, according to the scheme summarized in Figure 2C. Previous studies mention a basal level of *FUS1* expression in haploid cells, resulting from endogenous ligand-independent signaling via the MAP kinase cascade (Hagen *et al.* 1991; Chasse *et al.* 2006; Lee and Dohlman 2008), and the sequencing data from the screen clearly support this, as many of the corresponding MAP kinase knockout mutants show an “Un Gfp^–^” phenotype.

**Figure 3:**
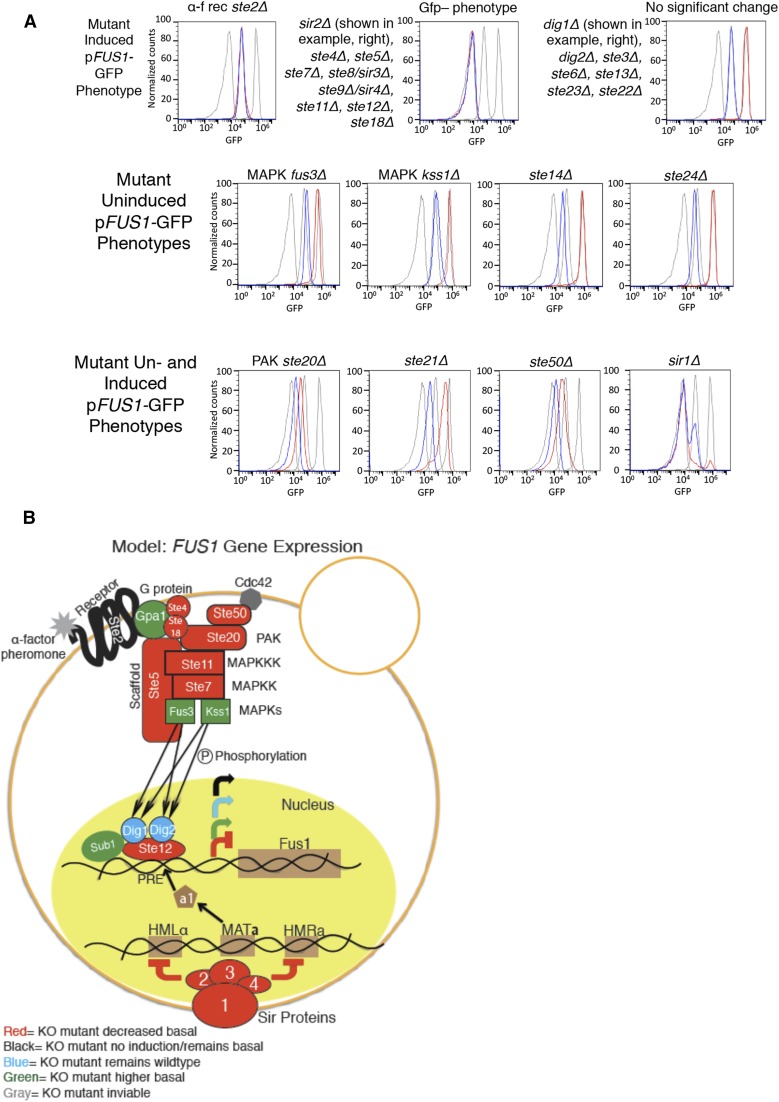
Known mutants in the mating pathway, and color-coded summary model. (A) *FUS1*-GFP of known mating pathway mutants. (B) Color-coded model of mutants in the pheromone response pathway based on mutant *FUS1*-GFP expression.

One expected class of mutants is those that lack p*FUS1*-GFP expression under both basal and α-factor-induced conditions (the “Un Gfp^–^” and “In Gfp^–^” mutants in Figure 2B, Figure 2C, Table S1, and Table S2). In this population, we observed an enrichment of mutants in genes integral to the MAPK pathway itself (Hagen *et al.* 1991), including the MAPKKK mutant *ste11*, MAPKK mutant *ste7*, the Gβ subunit mutant *ste4*, and the pheromone-responsive *ste5* scaffold protein mutant; indeed, these were all high-ranking mutants (Table S1 and Table S2). We also observed enrichment of the Silent Information Regulator (*sir*) mutants in the Gfp^–^ sorted pools. *Sir* mutants are expected among this class of mutants, as they have defects in silencing at the silent mating loci *HMR* and *HML*. Due to the consequent coexpression of *HMR***a** and *HML*α information, these mutants behave as diploids—they are defective in mating, and do not express haploid-specific genes, including *FUS1* (Rine and Herskowitz 1987) (Figure 3, Table S1, and Table S2).

Another class of mutants observed in our screen, the “In Gfp^basal^” population, includes those that have normal basal levels of p*FUS1*-GFP expression, but fail to induce p*FUS1*-GFP expression to wild-type levels upon induction with α-factor. In this category, we observed *ste2* mutants, defective for the G-protein-coupled receptor that, upon binding α-factor, pheromone signals through the downstream MAPK cascade that ultimately leads to an increase in expression of mating genes, including *FUS1* (Figure 1). The *ste2* mutants were highly enriched in the “basal” p*FUS1*-GFP sorted population following α-factor treatment (“In Gfp^basal^” population, Figure 2B, Figure 2C, and Table S3), corresponding to a pheromone-unresponsive phenotype as previously reported (Chasse *et al.* 2006).

After sorting, the mutant populations were separately expanded and stored as glycerol stocks. To evaluate the accuracy of our sorting regimen, these populations were subjected to another round of growth, with or without pheromone treatment, and run on a flow cytometer for FACS analysis to investigate how reproducibly the cell sorting separated the different populations of mutants based on fluorescence, according to the scheme in Figure 2B. The FACS analysis profiles of the sorted subpopulations after another round of growth largely recapitulated the GFP fluorescence intensities by which they were defined (Figure S2). The Gfp^–^ populations that were sorted (Figure S2, top panel) had very clean peaks upon FACS analysis after another round of growth. The other sorted populations (“In Gfp^basal^” in top panel, “Un Gfp^+^” in bottom panel and “In Gfp^++^” in bottom panel, Figure S2 were also enriched for the fluorescence levels that they were sorted into; however, these were less pure/reproducible than the Gfp^–^ sorted populations, probably because the number of mutants with this phenotype is very small, and/or was shifted only modestly in fluorescence intensity from the wild type.

### Mutants with higher basal FUS1 expression

In this study, we sorted for an interesting population: the “Un Gfp^+^” population, *i.e.*, cells with higher basal *FUS1*-GFP levels than wild type under vegetative growth conditions. This is a category of mutants that has not been studied in depth, in part because the ranked lists of mutants did not show good coherence and, consistent with this, the fluorescence profile of the sorted populations after an additional round of growth showed lower purity than that observed for the sorted Gfp^–^ populations after regrowth. We also investigated whether there were any mutants (the “In Gfp^++^” mutants) that displayed hyper-induction of the *FUS1*-GFP reporter under α-factor treatment. We observed that many of the top hits in these two populations overlap, and are present in both of these populations (Table S4 and Table S5), and the fluorescence profiles of the populations when rerun support this observation (Figure S2, bottom panel), so we retested the top genes individually. From this retesting, we found that one of these, *SUB1*, has increased basal levels p*FUS1*-GFP expression. Intriguingly, *SUB1* was previously reported to be involved in a pathway parallel to the mating pathway (the osmotic stress response pathway) (Rosonina *et al.* 2009). In a subsequent section, we provide detailed support for the involvement of *SUB1* in the pheromone response pathway.

### “Known mutant survey” using the FUS1-GFP reporter assay

The rankings of the mutants provided by deep sequencing of the barcodes gives an initial picture of the phenotypes of the knockout mutants using the single cell precision of flow cytometry analysis that is not afforded by other “bulk” reporter assays. The majority of previous studies looking at *FUS1* expression were done by looking at the total *FUS1*-lacZ level of a strain (bulk assays) (Chasse *et al.* 2006; Parrish *et al.* 2002; Bardwell *et al.* 1998; Posas and Saito 1997). To avoid potential complications resulting from genetic “liabilities” of the knockout library used, we generated newly made mutants of the known genes in the mating pathway, and evaluated the p*FUS1*-GFP fluorescence profile of each strain individually by flow cytometry (Figure 3A, Table S6a, and Table S6b). This method differs from the *FUS1*-lacZ technique previously used, in that the flow cytometry data records the fluorescence data of each individual cell, and gives a readout that reflects this. FACS data are more sensitive, and indicate whether all cells in the population of a strain have uniform *FUS1*-GFP expression or not. These data allowed us to build an updated model of the mating pathway by color-coding the different mutants based on their *FUS1*-GFP phenotype (Figure 3B), with the genes in the MAPK pathway color-coded to reflect the different p*FUS1*-GFP expression phenotypes. We also expand the model to include the components of the *SIR* complex, a1, and the MAT**a** locus, indicating their importance in maintaining haploid specificity, and thus haploid-specific expression of *FUS1* in *MAT****a*** cells.

Significantly, the role that some of the known “*sterile*” mutants play in *FUS1* expression is not fully understood. For example, *ste14*Δ was previously identified as having lower basal *FUS1* expression levels (Xu *et al.* 1998), which we also observe here; however, the biological rationale and mechanism of this have not yet been deciphered. *STE14* is a *MAT***a** haploid-specific gene, and encodes the farnesyl cysteine-carboxyl methyltransferase that functions in **a**-factor processing; it must have some role in signaling in *MAT***a** cells as well. Ste14 adds a methyl group to the carboxyl of all farnesylated, and some geranylgeranylated, proteins (Michaelis and Barrowman 2012). There are about 90 of these farnesylated and geranylgeranylated proteins, including many small GTPases, like Ras and Rho, which are important signaling molecules, as well as the γ subunit of heterotrimeric G proteins (like Ste18 of the mating G protein). Perhaps one of these Ste14 substrates plays a role in modifying other proteins that affect basal MAPK signaling. In support of this possible explanation, we observed that *MAT*α cells express lower levels of the p*FUS1*-GFP reporter compared to *MAT***a** cells (data not shown), similar to the decrease in p*FUS1*-GFP expression seen in the *ste14*Δ deletion mutant.

Significantly, using our method of analyzing the mutants containing the *FUS1*-GFP reporter using flow cytometry to analyze these mutants, as opposed to the previously used *FUS1*-lacZ methods, allowed us to assess whether the *FUS1*-GFP reporter expression is uniform in a population. Interestingly, the p*FUS1*-GFP fluorescence profile of the *sir1*Δ mutant appears to consist of two subpopulations: a main population of Gfp^–^ under basal growth conditions, but also a subpopulation within the strain that clearly has basal GFP levels that increase to wild-type induced GFP levels upon α-factor induction. This is consistent with prior work that indicates that, in *sir1* mutants, epigenetic switching occurs between silenced and nonsilenced states (Pillus and Rine 1989), and was missed by the previously used “bulk” *FUS1*-lacZ assays.

### sub1**Δ** mutant has higher basal *FUS1*-GFP expression

After retesting the *sub1*Δ mutant from the *MAT***a** library for *FUS1* expression, we found that the strain from the library had reproducibly higher *FUS1*-GFP levels under basal conditions. When we generated newly made *sub1*Δ mutants (derived from wild-type diploids by sporulation), and tested them for *FUS1* expression, we found that these newly made *sub1*Δ deletion mutants indeed have higher basal levels of *FUS1*, but the induced levels in the *sub1*Δ mutant are indistinguishable from wild type. The *SUB1* gene encodes a transcriptional coactivator that was first identified in a screen designed to identify genes that, when overexpressed, are able to rescue the cold sensitivity of a TFIIB R78H mutant (Knaus *et al.* 1996). Experiments have since shown that *SUB1* plays a positive role in a wide range of stages in transcription, including initiation (Wu *et al.* 1999), promoter melting (Sikorski *et al.* 2011), elongation (Garcia *et al.* 2012), and 3′ end formation (He *et al.* 2003). Additionally, *SUB1* has a human homolog, PC4 (positive coactivator 4) (Henry *et al.* 1996), with similar properties. Other recent studies have reported that Sub1 functions in diverse contexts, such as establishing the quiescent state in yeast (Acker *et al.* 2014), protecting transcription initiation sites from mutations (Lada *et al.* 2015), and as a negative regulator of sporulation (Gupta *et al.* 2015).

**Figure 4:**
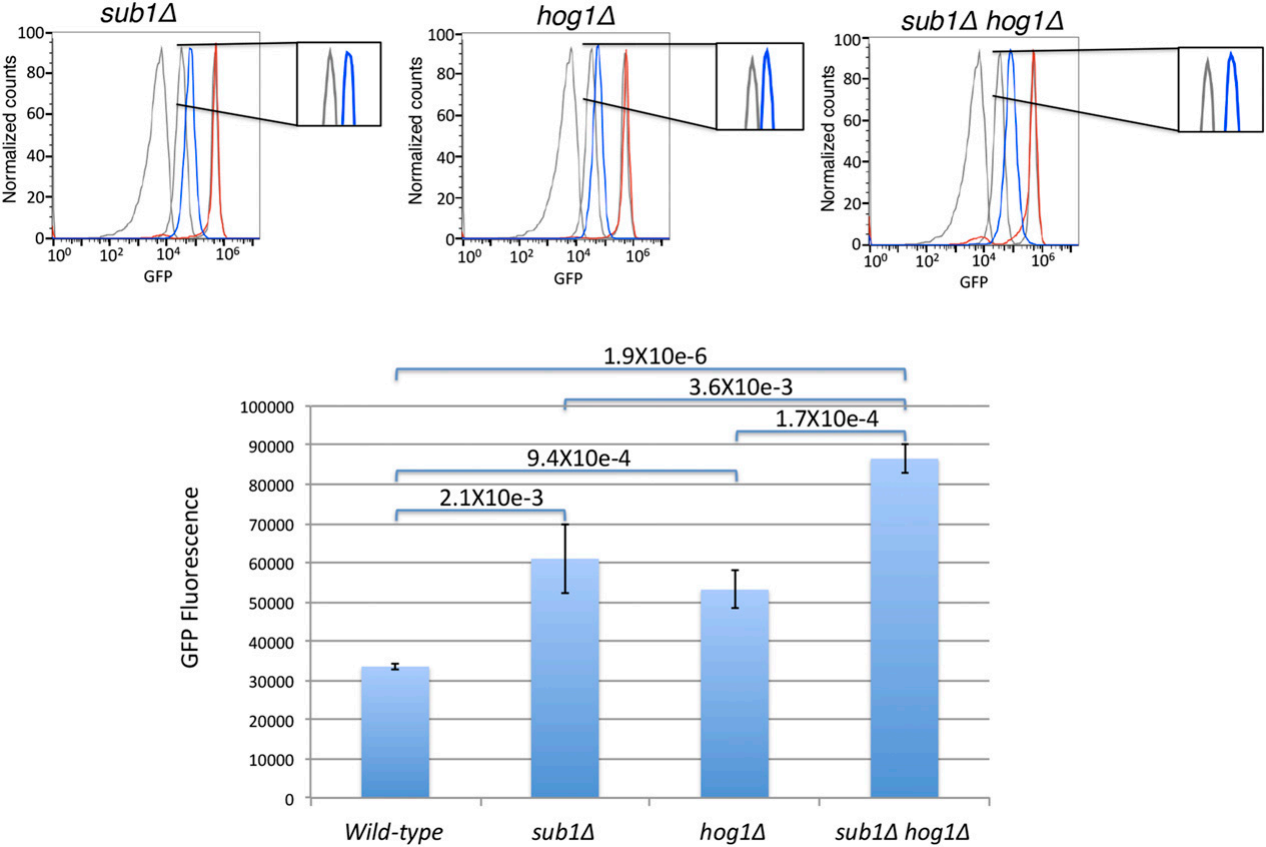
*sub1*Δ and *hog1*Δ mutants have higher p*FUS1*-GFP expression. *sub1*Δ and *hog1*Δ each have about a twofold higher basal *FUS1* expression than wild type; *sub1*Δ *hog1*Δ has about threefold higher basal level (wild type, three replicates; *sub1*Δ, five replicates; *hog1*Δ, 4 replicates; *sub1*Δ *hog1*Δ, three replicates).

Because of previous reports of *SUB1* and *HOG1* interactions (Rosonina *et al.* 2009) in the HOG (High Osmolarity Glycerol) pathway, we were interested to see how Hog1 and Sub1 together might influence *FUS1* expression. We generated newly made *hog1*Δ and *hog1*Δ *sub1*Δ mutants, and recapitulated the previous finding that the *hog1*Δ mutant also has a twofold increase of basal *FUS1* expression (Figure 4, plasmid rescue experiment in Figure S8, and Figure S9) (Hall *et al.* 1996) (similar to what we observed in a *sub1*Δ mutant). Additionally, we observed about a threefold increase in the basal expression of *FUS1* in the *hog1*Δ *sub1*Δ double mutant (Figure 4). Together, this suggests that *SUB1* and *HOG1* act via separate mechanisms to keep basal *FUS1* expression levels repressed in vegetative growth conditions. Intriguingly, although Sub1p clearly functions as a coactivator in *in vitro* experiments, it additionally functions in a pathway that limits expression of *FUS1*, as *sub1*Δ mutants have a mutant p*FUS1*-GFP phenotype similar to that of a *hog1*Δ mutant.

### RNA-Seq of sub1**Δ** mutant

**Figure 5:**
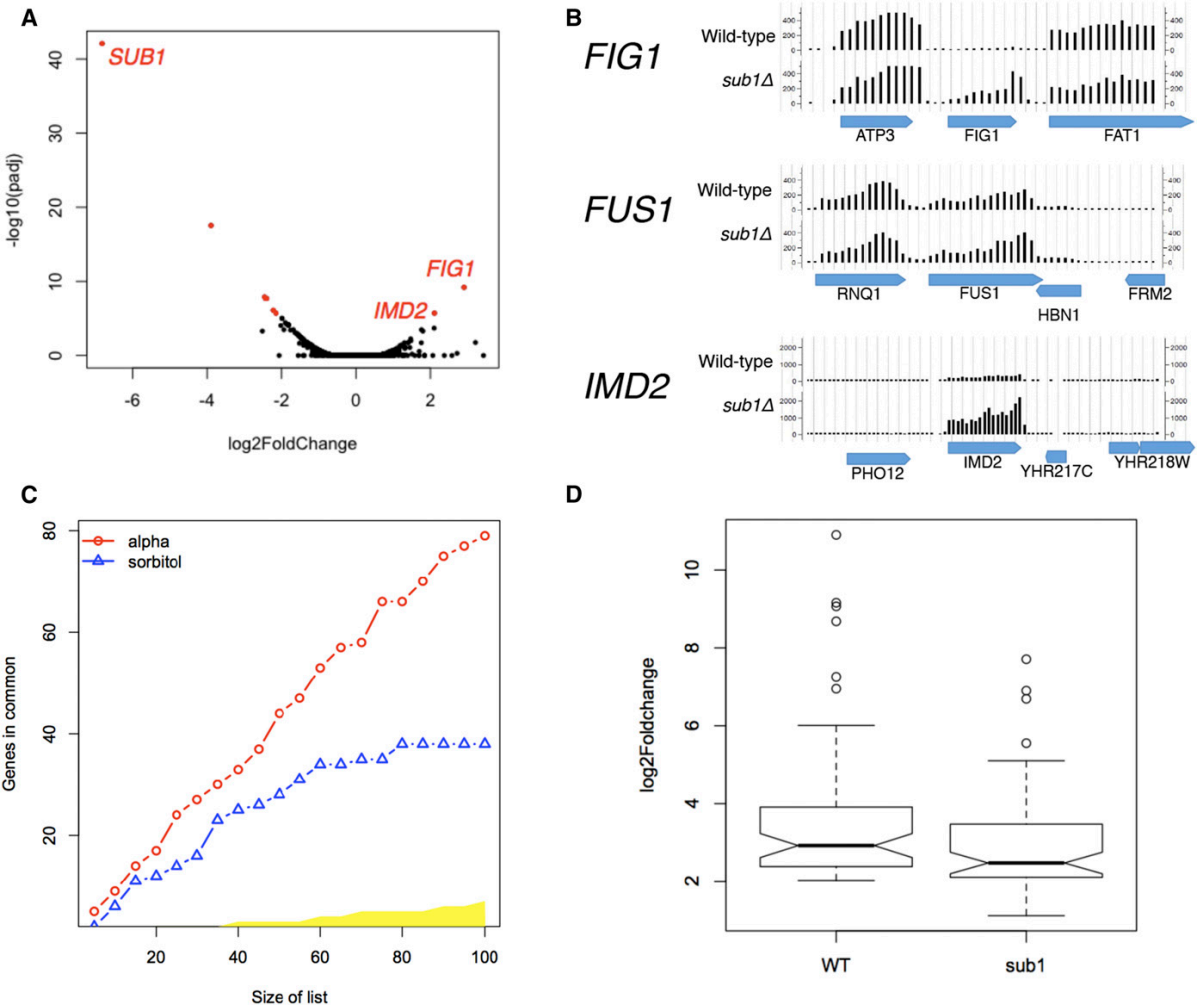
RNA-Seq of *sub1*Δ mutant. (A) Volcano plot: RNA-Seq in vegetative growth conditions of *sub1*Δ and wild type shows that most genes have unchanged expression; most genes with changed expression relative to wild type are left-shifted, consistent with an overall positive role of *SUB1* on transcription. (B) *FIG1* and *IMD2* have significantly higher basal levels in *sub1*Δ, *FUS1* has 1.2-fold higher basal levels in the *sub1*Δ mutant that, while not statistically significant, nevertheless agrees with the modest increase in GFP expression observed. (C) Concordance at the top (CAT) plot, showing that the RNA expression profile of a *sub1*Δ mutant in α-factor-induced conditions is indistinguishable from wild type, while the RNA expression profile of these two strains is significantly different in high sorbitol conditions (yellow area represents the 99.9% critical region for the null hypothesis of no correspondence). (D) Alpha-factor-inducible genes induce to a lower extent in a *sub1*Δ mutant compared to wild type, log2fold ≥ 2; padj < 0.01, about 90 genes.

To further identify genes regulated by *SUB1* under relevant growth conditions, we performed RNA-Seq on a *sub1*Δ mutant under vegetative growth, +α-factor, and +sorbitol conditions. There are several genes in the *sub1*Δ mutant that have either higher or lower gene expression in relation to the wild-type control under vegetative growth conditions (Figure 5A). Interestingly, and consistent with previous reports (Koyama *et al.* 2008), *IMD2* and related *IMD* genes are enriched among the genes that are, like FUS1 paradoxically (given Sub1’s demonstrated positive effects on *in vitro* transcription) more highly expressed in the *sub1*Δ mutant relative to the wild-type control (Table 1). Also consistent with previous reports (Koyama *et al.* 2008), our ChIP-Seq data supports that Sub1 binds the promoter of *IMD2* (data not shown). Significantly, similar to what we observed for *SUB1*, *FIG1* (a pheromone inducible gene that encodes for an integral protein necessary for efficient mating) is another gene that is also highly expressed in the *sub1*Δ mutant relative to the wild type in the absence of pheromone (Figure 5B). Consistent with the small increase in basal expression of *FUS1*-GFP phenotype we observed, we saw a modest 1.2-fold increase in RNA levels of *FUS1* in the *sub1*Δ mutant as assessed by read counts. Even though there are a small number of genes that show higher expression, the majority of dysregulated genes are skewed toward underexpression in the *sub1*Δ mutant, consistent with an overall function as an activator of transcription (Figure 5A). The genes with significantly lower RNA expression in the *sub1*Δ mutant are highly enriched for four GO-Slim Process terms: response to osmotic stress, response to heat stress, carbohydrate transport, and generation of precursor metabolites and energy genes (Table 2).

**Table 1:** Genes with higher basal expression in a *sub1***Δ** mutant

Systematic Name	Common Name	Log2 Fold Change	padj	Description
YBR040W	*FIG1*	2.90	6.43E-10	Integral membrane protein required for efficient mating
YHR216W	*IMD2*	2.11	1.95E-06	Inosine monophosphate dehydrogenase; catalyzes the rate-limiting step in GTP biosynthesis
YGL263W	*COS12*	2.10	0.000206	Protein of unknown function; member of the *DUP380* subfamily of conserved, often subtelomerically encoded proteins
YAR075W	*YAR075W*	1.80	0.000510	Nonfunctional protein with homology IMP dehydrogenase; *YAR073W/IMD1* and *YAR075W* comprise a continuous reading frame in most strains of *S. cerevisiae*
YAR073W	*IMD1*	1.759	0.000330	Nonfunctional protein with homology to IMP dehydrogenase; blocked reading frame, located close to the telomere; not expressed at detectable levels; *YAR073W/YAR075W* together have a paralog, *IMD2*, that arose from a segmental duplication
YER138W-A	*YER138W-A*	1.469	0.00576	Putative protein of unknown function; *YER138W-A* has a paralog, *YBL107W-A*, that arose from a single-locus duplication

This table shows the results from an RNA-Seq experiment. These are the genes that are more highly expressed in a *sub1*Δ mutant relative to wild type.

**Table 2:** GO analysis of genes with lower basal expression in a *sub1***Δ** mutant

GO-Slim Term	Cluster Frequency	Genome Frequency	Genes Annotated to the Term	padj Value
Response to osmotic stress	8 out of 44 genes, 18.2%	91 of 6338 genes, 1.4%	ALD6, CIN5, HSP12, MRK1, MSN4, SUB1, USV1, XBP1	1.61E-05
Generation of precursor metabolites and energy	7 out of 44 genes, 15.9%	159 of 6338 genes, 2.5%	*GAC1, GLC3, GPH1, HAP4, HXK1, ISF1, RGI2*	0.00317
Carbohydrate transport	6 out of 44 genes, 13.6%	33 of 6338 genes, 0.5%	*HXK1, HXT2, HXT4, HXT6, HXT7, MTH1*	1.61E-05
Response to heat	6 out of 44 genes, 13.6%	69 of 6338 genes, 1.1%	*DDR2, GAC1, HSP12, MRK1, MSN4, XBP1*	0.000448

This table shows that the genes with lower basal transcription in a *sub1*Δ mutant compared to a wild-type strain are enriched for four GO-Processes (data from an RNA-Seq experiment).

While the RNA expression profile in wild type and the *sub1*Δ mutant are very similar under vegetative growth conditions, we were interested to see whether Sub1 is necessary for the induction of pheromone inducible genes, and HOG inducible genes, under α-factor and sorbitol conditions, respectively. Upon further analysis of the RNA-Seq data, we observed that the transcriptional response of *sub1*Δ to α-factor resembles that of wild type, while the induction of genes in response to high osmolarity in the *sub1*Δ mutant is clearly distinct from that of wild type [see “Concordance At the Top” (CAT) plot, Figure 5C], consistent with Sub1 being required for induction of a specific set of genes under high sorbitol conditions. Significantly, on further computational analysis, we observe that, even though the pheromone-inducible genes are induced in a *sub1*Δ mutant, as a group they induce to substantially lower level than wild type (Figure 5D).

### ChIP-Seq of Sub1-3HA

We tagged native Sub1 with a C-terminal 3HA tag, and performed a ChIP-Seq experiment to determine where in the genome Sub1 binds, similar to previous reports: Tavenet *et al.* 2009 performed a ChIP-chip experiment on 3HA-tagged Sub1 and observed Sub1 binding at 991 loci. From the ChIP-chip data, this latter group found that Sub1 was enriched for binding at Pol III-transcribed genes, and *in vitro* reconstitution assays showed that Sub1 stimulated transcriptional initiation and reinitiation. In additional *in vitro* assays, they observed that *in vitro* Pol III-transcribed RNA transcripts are greatly reduced when using the cell extracts from *sub1*Δ cells compared to when the cell extracts from wild-type controls were used.

**Figure 6:**
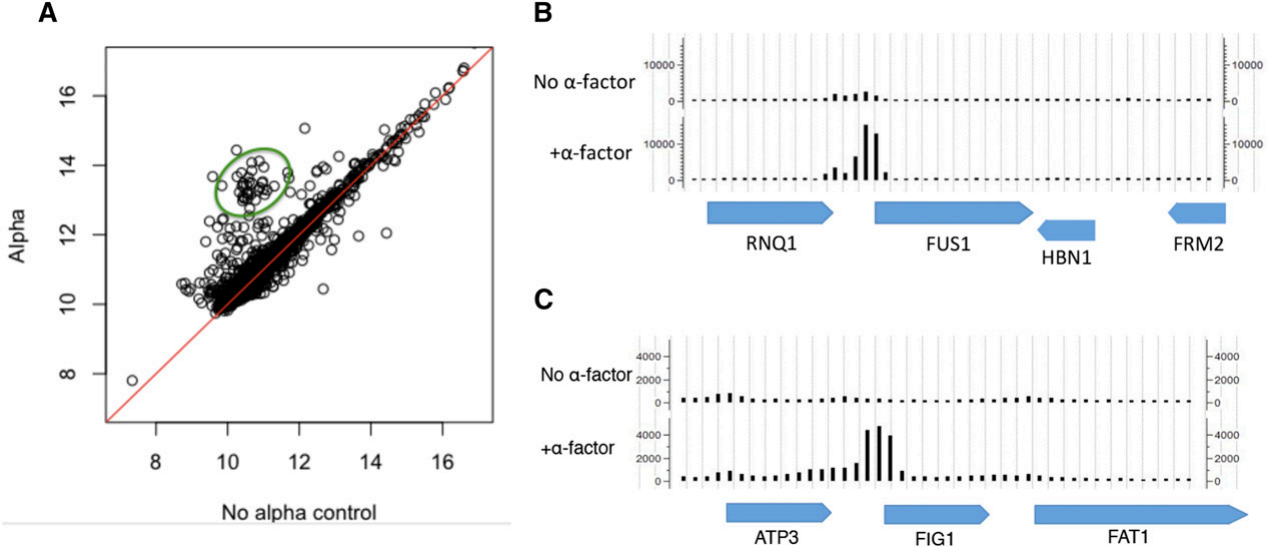
ChIP-Seq of Sub1 in α-factor induced conditions. (A) Sub1 exhibits increased binding at the promoters of ∼76 pheromone induced genes (circled in green). This plot was generated by first selecting peaks that increased with p-value < 0.05. Then the genes near these peaks were identified. (B) Sub1 increases binding at the promoter of *FUS1* upon α-factor induction. (C) Sub1 increases binding at the promoter of *FIG1* upon α-factor induction.

Here, we investigated where in the genome Sub1 binds under vegetative growth, α-factor, and high sorbitol conditions, and the ChIP-Seq data supports chromatin binding activity of Sub1 in all three conditions. We observed that Sub1 bound at an even higher number of genes than previously reported (we observed Sub1 binding at promoter regions of ∼3000 genes, about half of the genes in the genome) under vegetative growth conditions, supporting previous findings that Sub1 plays a general role in transcription. Our ChIP-Seq data also shows that, when induced by α-factor, Sub1 maintains binding at loci throughout the genome (Figure S3), and substantially increases binding to the promoters of a subset of ∼76 genes including *FUS1* (Figure 6A and Table 3). Upon α-factor treatment, Sub1 significantly increases binding to the promoters of *FUS1* and *FIG1* (Figure 6B and Figure 6C). Significantly, and consistent with the involvement of *SUB1* in the mating pathway, we found that the genes with higher Sub1 binding levels under α-factor conditions are GO-enriched for conjugation, organelle fusion, nuclear organization, cell morphogenesis, and transposition genes (Kurihara *et al.* 1996; Erdman *et al.* 1998). Together, these data support the involvement of *SUB1* in the pheromone response.

**Table 3:** ChIP-Seq of Sub1-3HA under α-factor induction

GO-Slim Term	Cluster Frequency	Genome Frequency	Genes Annotated to the Term	padj Value
Conjugation	13 out of 76 genes, 17.1%	125 of 6338 genes, 2%	*AFR1, ASG7, CIK1, FIG1, FIG2, FUS2, KAR3, KAR4, KAR5, PRM1, PRM2, PRM3, RVS161*	3.13E-07
Transposition	8 out of 76 genes, 10.5%	110 of 6338 genes, 1.7%	*YDR098C-A, YDR098C-B, YGR109W-A, YGR109W-B, YHL009W-A, YHL009W-B, YIL082W, YIL082W-A*	0.00151
Organelle fusion	8 out of 76 genes, 10.5%	88 of 6338 genes, 1.4%	*CIK1, FUS2, KAR3, KAR4, KAR5, PRM2, PRM3, VTI1*	0.000557
Nucleus organization	7 out of 76 genes, 9.2%	64 of 6338 genes, 1.0%	*CIK1, FUS2, KAR3, KAR4, KAR5, PRM2, PRM3*	0.000557
Cell morphogenesis	4 out of 76 genes, 5.3%	29 of 6338 genes, 0.5%	*AFR1, FIG1, FIG2, FUS2*	0.0138

Genes enriched for Sub1 binding under +α-factor (from SGD GO-Slim: Process).

Even more strikingly, from the ChIP-Seq data, we also observe that Sub1 remained bound at only a subset of genes upon treatment with sorbitol (Figure S3). This observation is consistent with a previous study (Proft and Struhl 2004), where hyperosmotic stress was reported to cause a rapid dissociation of three transcriptional regulator proteins: Gal4, Rap1, and Sko1 (while histones and elongating Pol II remained bound to chromatin under hyperosmotic conditions). The authors of this paper speculate that the sudden shift to high osmolarity causes a rapid increase of ionic strength in the nucleus, which then leads most chromatin-associated proteins to dissociate from the DNA.

## Discussion

The screening method developed here identified many of the known yeast mating pathway genes and additionally discovered a novel function of *SUB1*, a gene that affects basal transcription of *FUS1*. As such, our screening method of large-scale transformation of the YKO library pool, followed by FACS sorting and Bar-Seq, may be useful in future studies to better understand other less well-characterized dynamic systems.

As with other screens, the design of this pooled screen does have some limitations that lead to false positives Supplemental Text, Figure S6, and Figure S7. One limitation of this particular screen is that the YKO collection used is haploid, and thus we expect that mutants conferring slow growth and lethal phenotypes will be underrepresented and absent from this screen, respectively. For example, the *ste16*Δ and *gpa1*Δ mutations in haploids confer slow growth and lethal phenotypes, respectively, (Wilson and Herskowitz 1987; Miyajima *et al.* 1987) and, as expected, these mutants were not identified in this screen. Thus, in future screens using the YKO library, it will continue to be important to consider the limitations as well as possible sources of false positives and false negatives that may arise from the design of the screen.

The “known mating pathway mutant” portion of this study highlights that there are many genes with positive, negative, or insulating functions in the MAPK mating pathway, that have mutant p*FUS1*-GFP reporter expression when knocked out, and our sensitive assay of measuring the *FUS1*-GFP fluorescence by flow cytometry allows us to detect subtle changes at the single cell level. Mutants in the mating pathway that have redundant functions (*dig1*Δ and *dig2*Δ), function in pheromone processing, or export (ste6Δ, *ste22*Δ, and *ste23*Δ), or function in *MAT*α cells (*ste3*Δ and *ste13*Δ) have p*FUS1*-GFP expression that is indistinguishable from wild type. The *ste2*Δ mutant has wild-type basal levels of p*FUS1*-GFP but fails to induce expression upon induction with α-factor, consistent with basal activity of the *FUS1* promoter dependent on the MAPK pathway. Mutants with Gfp^–^ expression include those integral to the MAPK pathway itself (*ste4*Δ, *ste5*Δ, *ste7*Δ, *ste11*Δ, *ste12*Δ, and ste18Δ), and the *sir* mutants (*sir2*Δ, *sir3*Δ, and *sir4*Δ). As discussed above, the *sir1*Δ mutant has two subpopulations, a main Gfp^–^ subpopulation, and a small population with a wild-type level of basal GFP that induces to wild-type levels upon induction with α-factor. The MAPK mutants *fus3*Δ and *kss1*Δ have higher basal p*FUS1*-GFP expression, consistent with previous reports (Bardwell *et al.* 1998; Hall *et al.* 1996). The *ste14*Δ (discussed in the *Results* section), and *ste24*Δ mutants also have lower basal *FUS1*-GFP expression compared to wild type. Ste24 cleaves the AAX off some (but not all) farnesylated CAAX proteins (Michaelis and Barrowman 2012). This cleavage must occur for Ste14 to add the methyl group, so it is not surprising that the basal *FUS1* expression is lower for both mutants. It will be interesting to determine whether this effect is due to the lack of methyl modification of one particular protein (perhaps Ste18m, the γ subunit of the G protein involved in mating); it may be that the lack of methyl modification may affect its activity negatively. The PAK *ste20*Δ mutant, and adaptor protein *ste50*Δ mutant, also have very low basal and induced *FUS1*-GFP expression compared to wild type, as expected (Chasse *et al.* 2006). The *ste21*Δ karyopherin mutant has both lower basal and induced *FUS1*-GFP levels (Blondel *et al.* 1999). Significantly, the “known mutant survey” of this study highlights that there are many genes that affect the expression of *FUS1*, many of them are subtle and some genes, including *SUB1*, have not been previously identified.

We identified *sub1*Δ, which exhibits increased levels of the p*FUS1*-GFP reporter. The apparent repressive effect of *SUB1* on basal expression of *FUS1* is paradoxical, given the numerous studies suggesting that it acts as a transcriptional activator *in vitro*, and our own work showing that the majority of affected genes in the *sub1* mutant go down in expression rather than up. How can this paradox be rationalized? Interestingly, consistent with our observation that *sub1*Δ has increased basal levels of *FUS1* compared to wild type, one previous study suggested a repressive function for *SUB1* (Koyama *et al.* 2008), in which *SUB1* was reported to function in the repression of *IMD2*. In a wild-type cell under vegetative growth conditions, *IMD2* transcripts are made from the upstream transcription start site, and short transcripts from transcriptional termination result in repression of *IMD2*. In a *sub1*Δ mutant under vegetative growth conditions, the authors observe an increase in full-length *IMD2* transcripts (which we confirmed), and a decrease in the short *IMD2* transcripts, their data suggesting that *SUB1* may play a role in transcription start site selection. In further studies, it will be interesting to see if Sub1 is involved in producing short transcripts of *FUS1*, similar to the mechanism of short transcripts in *IMD2*. However, there was no clear-cut evidence for this from the RNA-Seq data, perhaps because we evaluated only a polyA-enriched RNA fraction, and thus we sought other potential explanations for the paradoxical effect on *FUS1* expression.

In yeast, there are five MAPK pathways that function in distinct processes, several components of which are shared between the parallel pathways (Levin and Errede 1995). The expression of two other genes in MAPK pathways parallel to the pheromone response pathway (*MSN4* and *TEC1*) is lower in the *sub1*Δ mutant, as is the case for most *SUB1* targets. *MSN4* encodes a transcription factor that regulates genes in response to osmotic shock stress, while *TEC1* encodes a transcription factor in the filamentation pathway that controls cell shape and biofilm formation. The transcription factor from the pheromone response pathway, Ste12, forms a complex with Tec1, and this complex regulates a number of filamentation genes. We therefore hypothesize that, since these MAPK pathways share a number of MAPK components, it is possible that, once expression of a component of one pathway is reduced, the shared MAPK components (*e.g.*, Ste12 protein) will now be freed up to contribute to a greater degree in the pheromone response pathway, thus increasing the basal expression of genes in the pathway, including *FUS1*.

Here, in the first ChIP-Seq of Sub1, we show that Sub1 exhibits global binding to genes in the genome, and thus likely plays a supporting role in transcription of most of these genes, and to fine-tune their expression. The ChIP-Seq data here tripled the number of genes Sub1 binds to (compared to a previous ChIP-chip study). Additionally, in this study we perform the first RNA-Seq of a *sub1*Δ mutant, and we show that *SUB1* clearly affects the expression of a small number of genes that are involved in various stress responses, furthering knowledge of the genes regulated by Sub1. The RNA-Seq data are consistent with previous studies, where *SUB1* was reported to play a distinct role in various stress conditions, including high osmolarity, DNA damage, and reentering the cell proliferation state. Here we show that *SUB1* is involved in the dynamic system of pheromone response, and contributes to the expression of genes in the pheromone response pathway.

## 

## Supplementary Material

Supporting Information
