# Supplementary material for: Barcode Sequencing Screen Identifies SUB1 as a Regulator of Yeast Pheromone Inducible Genes
Source: G3 (Bethesda). 2016 Feb 1;6(4):881–92. doi: 10.1534/g3.115.026757 (PMC4825658; doi:10.1534/g3.115.026757)
Supplement: Supporting Information [file supp_6_4_881__index.html]

Barcode Sequencing Screen Identifies SUB1 as a Regulator of Yeast Pheromone Inducible Genes — Supporting Information 

# Barcode Sequencing Screen Identifies *SUB1* as a Regulator of Yeast Pheromone Inducible Genes

## Supporting Information for Sliva *et al.*, 2016

**Files in this Data Supplement:**

- Figure S1 - The wild-type strain transformed with the reporter construct (yAS38 control strain) forms shmoos and has higher GFP fluorescence under α-factor treatment. (.pdf, 432 KB)
- File S1 - Evaluation of individual mutants: A source of false positives. (.pdf, 343 KB)
- File S2 - Supplemental methods. (.pdf, 337 KB)
- Table S1 - Top genes enriched in "Un Gfp-" population. (.pdf, 360 KB)
- Table S2 - Top genes enriched in the "In Gfp-" population. (.pdf, 357 KB)
- Table S3 - Top genes enriched in "In Gfpbasal" population. (.pdf, 352 KB)
- Table S4 - Top genes enriched in "Un Gfp+" population. (.pdf, 350 KB)
- Table S5 - Top genes enriched in "In Gfp++" population. (.pdf, 350 KB)
- Table S6 - GFP fluorescence values of mating mutants in Figure 3. (.pdf, 717 KB)
- Table S7 - Strains used in this study. (.pdf, 442 KB)
- Figure S2 - FACS analysis of populations after sorting. (.pdf, 549 KB)
- Figure S3 - Screenshot of Sub1-3HA ChIP-Seq under high osmolarity conditions. (.pdf, 388 KB)
- Figure S4 - Gating of YKO library sorting experiments. (.pdf, 338 KB)
- Figure S5 - SUB1-3HA tagged strain retains some Sub1 function. (.pdf, 285 KB)
- Figure S6 - *whi3 Δ* and *kch1 Δ* mutants from the *MAT*a YKO library are diploid. (.pdf, 424 KB)
- Figure S7 - Fluorescence peak of YKO library shifts with a higher concentration of α-factor. (.pdf, 382 KB)
- Figure S8 - Plasmid rescue of *sub1 Δ* mutant. (.pdf, 425 KB)
- Figure S9 - *FUS1* expression is higher in a *sub1 Δ* mutant relative to wild-type. (.pdf, 358 KB)
