# Supplementary material for: Barcode Sequencing Screen Identifies SUB1 as a Regulator of Yeast Pheromone Inducible Genes
Source: G3 (Bethesda). 2016 Feb 1;6(4):881–92. doi: 10.1534/g3.115.026757 (PMC4825658; doi:10.1534/g3.115.026757)
Supplement: Supporting Information [file supp_g3.115.026757_FigureS1.pdf]

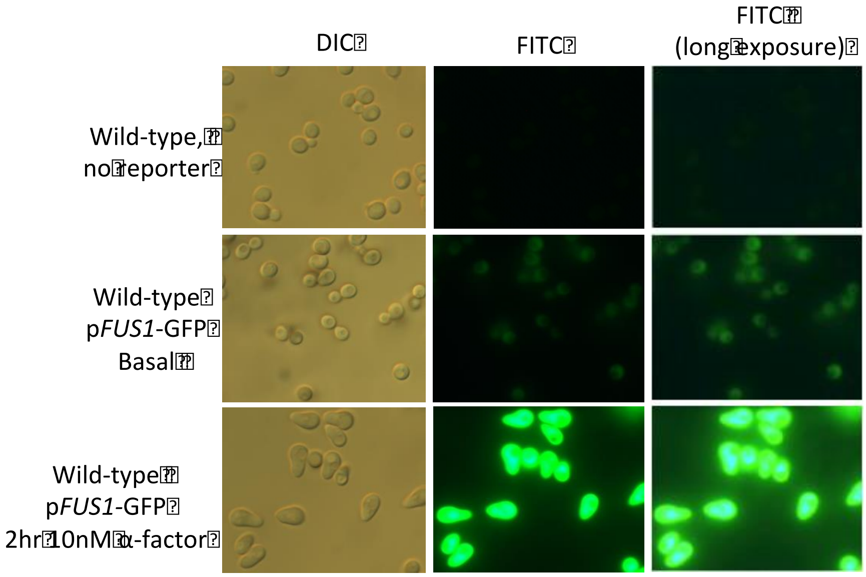

Figure S1 The wild-type strain transformed with the reporter construct (yAS38 control strain) forms shmoos and has higher GFP fluorescence under α-factor treatment.
