# Supplementary material for: Barcode Sequencing Screen Identifies SUB1 as a Regulator of Yeast Pheromone Inducible Genes
Source: G3 (Bethesda). 2016 Feb 1;6(4):881–92. doi: 10.1534/g3.115.026757 (PMC4825658; doi:10.1534/g3.115.026757)
Supplement: Supporting Information [file supp_g3.115.026757_FileS1.pdf]

Supplemental Text: Evaluation of individual mutants: A source of false positives

We tested candidate strains individually for mutant p*FUS1*-GFP expression patterns. The candidate deletion strains from the *MAT***a** YKO collection were transformed with the reporter construct and assayed for p*FUS1*-GFP expression individually (Tables S1-S5). From this individual retesting, we observed a number of false positive hits from the screen. This may be due to a number of reasons. The p*FUS1*-GFP reporter used in this screen was designed to integrate at the *BAR1* locus thus knocking out Bar1 function, rendering these cells more sensitive to α-factor while simultaneously introducing the reporter. Even though the majority of Leu^+ transformants had correct integration of the reporter knocking out *BAR1*, a small percentage (3%) were $Bar1^+$ and Leu^+, suggesting that the construct must have integrated elsewhere in the genome. These cells with intact Bar1 would require higher amounts of α-factor for wild-type *FUS1*-GFP induction, and thus these cells would not induce to wild-type *FUS1*-GFP levels at the level of α-factor used in the screen. Another explanation is that some of these mutants may have pre-existed in the pool as *MAT***a**/*MAT***a** diploids and thus had two copies of *BAR1* to begin with, as discussed further below, which would render them, unlike the rest of the library, Bar^+ after reporter integration.

We further investigated the involvement of two mutants that were highly enriched in the sorted "In Gfp^{basal}" population, *whi3Δ* (encoding for an RNA binding protein involved in the regulation of genes involved in the cell cycle) and *kch1Δ* (encoding for a potassium transporter involved in mediating the influx of potassium

and activating the high-affinity Calcium influx system during the pheromone response). Upon individual retesting, the *whi3Δ* and *kch1Δ* mutants clearly had a mutant *FUS1*-GFP expression phenotype, similar to what we would expect to see if functional Bar1 was present. Therefore, we evaluated ploidy of these strains by more definitive assays. When these cells were replica-plated to canavanine plates, the *whi3Δ* and *kch1Δ* strains from the *MAT***a** library did not form canavanine resistant papillae as expected for haploid cells (Schild et al. 1981); instead the cells were canavanine sensitive, consistent with the phenotype of diploid strains (Figure S6A). We also stained these cells with propidium iodide and by flow cytometry analysis, observed that their DNA content was consistent with the DNA content of a 2N diploid copy number (Figure S6B).

Next, we generated newly made diploid *WHI3/whi3Δ* and *KCH1/kch1Δ* mutants by transforming BY4743 containing the p*FUS1*-GFP reporter with the *whi3Δ* and *kch1Δ*::kanMX cassettes. After sporulating and dissecting the tetrads, we observed that the *kch1Δ* mutant has a wild-type phenotype under both "Un" and "In" conditions, while the *whi3Δ* mutant has lower basal and lower induced levels of *FUS1* expression relative to wild-type.

Altogether, these results support the hypothesis that the *whi3Δ* and *kch1Δ* mutants in the starting pooled *MAT***a** library used for the screen were in fact *MAT***a**/*MAT***a** diploids. This is consistent with previous reports of the haploid YKO library, where diploidization of certain mutants has been observed, usually in mutants affecting cell cycle and mitotic progression (Giaever and Nislow 2014). In this screen, this issue of diploidization prior to incorporation of the reporter

cassette is of particular importance, since when the reporter was transformed into these cells it would be present, but in a heterozygous state, leaving one functional copy of *BAR1*. Expression of functional Bar1 protein results in cleavage of α-factor, ultimately requiring higher amounts of α-factor for a fully wild-type pheromone response. This small percentage of $Bar1^{+}$ cells in the YKO pool also likely explains why the peak of the induced transformed YKO pool has slightly lower overall fluorescence than the WT control α-factor induced population (Figure 2C). Adding a higher concentration of α-factor (100nM) to the transformed YKO library shifts the peak of GFP fluorescence of the library to wild-type levels (Figure S7). In addition to the fact that multiple strains in the YKO pool started off as *MAT***a**/*MAT***a** diploids, it is well known that one of the components of the yeast transformation mix (PEG) can cause adjacent haploid cells to fuse during the transformation process, resulting in diploid cells (Svoboda 1978).
