# Supplementary material for: Barcode Sequencing Screen Identifies SUB1 as a Regulator of Yeast Pheromone Inducible Genes
Source: G3 (Bethesda). 2016 Feb 1;6(4):881–92. doi: 10.1534/g3.115.026757 (PMC4825658; doi:10.1534/g3.115.026757)
Supplement: Supporting Information [file supp_g3.115.026757_FileS2.pdf]

**Supplemental Methods:** Primers used in this study. (The specific multiplex tag sequences used are listed in the GEO data submission file).

## UPTAG Primer Sequences

(Illumina Adaptor sequence, unique multiplex tag, U1):
AATGATACGGCGACCACCGAGATCTACACTCTTTCCCTACACGACGCTCTTCCGATCTxxx GATGTCCACGAGGTCTCT

U1 UPTAG primers above were used with a modified U2c* kanMX sequence:
CAAGCAGAAGACGGCATACGAGCTCTTCCGATCTtggatgcaaatgattatacatgg (Illumina Adaptor sequence, kanMX sequence) to amplify the YKO UPTAG barcodes from each sorted population. The size of the amplicons from this PCR reaction is ~350bp.

## DNTAG Primer Sequences

(Illumina Adaptor sequence, unique multiplex tag, D1)

AATGATACGGCGACCACCGAGATCTACACTCTTTCCCTACACGACGCTCTTCCGATCTxxx CGGTGTCGGTCTCGTAG

D1 DNTAG primers above were amplified with a modified D2c* sequence:
CAAGCAGAAGACGGCATACGAGCTCTTCCGATCTtaacgccgccatccagtgtcg (Illumina Adaptor sequence, *kanMX* sequence) to amplify the YKO DNTAG barcodes from each sorted population. The size of the amplicon from this PCR reaction is ~180bp.

## References


Giaever G, Nislow C. 2014. The yeast deletion collection: a decade of functional genomics*. Genetics* **197**: 451-465.

Schild D, Ananthaswamy HN, Mortimer RK. 1981. An endomitotic effect of a cell cycle mutation of Saccharomyces cerevisiae*. Genetics* **97**: 551-562.

Svoboda A. 1978. Fusion of yeast protoplasts induced by polyethylene glycol*. J Gen Microbiol* **109**: 169-175.
