# Supplementary material for: Barcode Sequencing Screen Identifies SUB1 as a Regulator of Yeast Pheromone Inducible Genes
Source: G3 (Bethesda). 2016 Feb 1;6(4):881–92. doi: 10.1534/g3.115.026757 (PMC4825658; doi:10.1534/g3.115.026757)
Supplement: Supporting Information [file supp_g3.115.026757_TableS1.pdf]

Table S1. Top genes enriched in "Un Gfp–" population.
Genes in orange are expected to be enriched in this population; YDL041W* overlaps completely with *SIR2/YDL042C;* N/A = slow growth or lethal phenotype; not tested for p*FUS1*-GFP expression.

Systematic Name	Common Name	Log2 Fold Change	padj	Individually Tested Phenotype	Newly Made Mutant Phenotype
**YPR126C**	YPR126C	9.427	4.37E-05	Gfp⁻; no induction	Wild-type
**YLR442C**	SIR3	9.324	1.40E-08		Gfp⁻; no induction
**YDL159W**	STE7	9.283	2.65E-05		Gfp⁻; no induction
**YDR227W**	SIR4	9.269	2.71E-14		Gfp⁻; no induction
**YIL011W**	TIR3	9.223	1.74E-06	Wild-type	
**YDL042C**	SIR2	9.177	1.74E-06		Gfp⁻; no induction
**YOR212W**	STE4	9.158	5.52E-05		Gfp⁻; no induction
**YDR103W**	STE5	9.153	4.46E-06		Gfp⁻; no induction
**YDR443C**	SSN2	9.111	4.87E-05	Wild-type	
**YDL041W***	YDL041W*	9.102	2.74E-08		
**YDR462W**	MRPL28	9.063	0.0009	Wild-type	
**YDL074C**	BRE1	9.041	1.71E-05		Wild-type
**YHR177W**	YHR177W	9.028	2.59E-05	Lower basal; no induction	Wild-type
**YOR359W**	VTS1	9.014	2.65E-05	Wild-type	
**YIL042C**	PKP1	9.000	1.35E-05	Wild-type	
**YBR085W**	AAC3	8.984	1.87E-08	Lower basal; no induction	Wild-type
**YIL066C**	RNR3	8.981	0.0002	Wild-type	
**YIL133C**	RPL16A	8.975	0.0020	Wild-type	
**YIL009W**	FAA3	8.973	0.0011	Wild-type	
**YIL012W**	YIL012W	8.966	0.0001	Gfp⁻; no induction	Wild-type
**YOR369C**	RPS12	8.962	5.25E-09	Lower basal; no induction	N/A
**YIL044C**	AGE2	8.961	1.99E-05		
**YLL026W**	HSP104	8.952	0.0004	Wild-type	
**YIL093C**	RSM25	8.945	6.29E-05	Gfp⁻; no induction	
**YIL032C**	YIL032C	8.942	1.80E-05	Wild-type	
**YIR013C**	GAT4	8.933	0.0002	Wild-type	
**YIL154C**	IMP2	8.924	0.0001	Gfp⁻; no induction	Wild-type
**YDL073W**	YDL073W	8.917	2.65E-05	Wild-type	
**YMR088C**	VBA1	8.913	2.50E-05		
**YNL264C**	PDR17	8.819	4.06E-06	Gfp⁻; no induction	Wild-type
**YIL014W**	MNT3	8.816	1.22E-05	Wild-type	
**YIL079C**	AIR1	8.815	0.0004	Wild-type	
**YHR178W**	STB5	8.811	2.79E-05	Wild-type	

**YIL025C**	YIL025C	8.717	0.0029	Wild-type	
**YBR032W**	YBR032W	8.679	0.0002	Lower basal	Wild-type
**YNR075W**	COS10	8.668	0.0037	Wild-type	
**YIL030C**	SSM4/DOA 10	8.632	6.60E-06	Lower basal	Lower basal
**YOR320C**	GNT1	8.537	8.09E-05		
**YNL288W**	CAF40	8.354	0.0193		
**YNR013C**	PHO91	8.328	0.0001		
**YKR101W**	SIR1	8.231	0.0047		Mixed population, most Gfp^{-} (See Figure 3)
