# Supplementary material for: Barcode Sequencing Screen Identifies SUB1 as a Regulator of Yeast Pheromone Inducible Genes
Source: G3 (Bethesda). 2016 Feb 1;6(4):881–92. doi: 10.1534/g3.115.026757 (PMC4825658; doi:10.1534/g3.115.026757)
Supplement: Supporting Information [file supp_g3.115.026757_TableS2.pdf]

Table S2. Top genes enriched in the "In Gfp⁻" population.
Genes in orange are expected to be enriched in this population; YDL041W* overlaps completely with *SIR2/YDL042C;* N/A = slow growth or lethal phenotype and not tested for p*FUS1*-GFP expression.

Systematic Name	Common Name	Log2 Fold Change	padj	Individually Tested Phenotype	Newly Made Mutant Phenotype
**YIL011W**	TIR3	9.885	0.0026	Wild-type	
**YDR227W**	SIR4	9.864	0.0026		Gfp⁻; no induction
**YIL030C**	SSM4/DOA10	9.817	0.0026	Lower basal	Lower basal
**YPR126C**	YPR126C	9.761	0.0026	Gfp⁻; no induction	Wild-type
**YDL073W**	YDL073W	9.729	0.0026	Wild-type	
**YIL032C**	YIL032C	9.720	0.0026	Wild-type	
**YBR085W**	AAC3	9.706	0.0026	Lower basal; no induction	Wild-type
**YNL264C**	PDR17	9.703	0.0026	Gfp⁻; no induction	Wild-type
**YIL042C**	PKP1	9.683	0.0026	Wild-type	
**YIL014W**	MNT3	9.682	0.0120	Wild-type	
**YLR442C**	SIR3	9.670	0.0047		Gfp⁻; no induction
**YDR103W**	STE5	9.639	0.0055		Gfp⁻; no induction
**YDR462W**	MRPL28	9.629	0.0055	Wild-type	
**YDR443C**	SSN2	9.626	0.0026	Wild-type	
**YIL066C**	RNR3	9.606	0.0026	Wild-type	
**YOR359W**	VTS1	9.600	0.0026	Wild-type	
**YIL009W**	FAA3	9.594	0.0026	Wild-type	
**YDL159W**	STE7	9.585	0.0049		Gfp⁻; no induction
**YIL044C**	AGE2	9.581	0.0026		
**YHR177W**	YHR177W	9.578	0.0026	Lower basal; no induction	Wild-type
**YIL154C**	IMP2	9.557	0.0028	Gfp⁻; no induction	Wild-type
**YIL012W**	YIL012W	9.552	0.0031	Gfp⁻; no induction	Wild-type
**YDL074C**	BRE1	9.548	0.0026		Wild-type
**YIL093C**	RSM25	9.546	0.0026	Gfp⁻; no induction	
**YHR178W**	STB5	9.545	0.0065	Wild-type	
**YDL041W***	YDL041W*	9.542	0.0026		
**YOR212W**	STE4	9.536	0.0049		Gfp⁻; no induction
**YIR013C**	GAT4	9.531	0.0026	Wild-type	
**YIL079C**	AIR1	9.523	0.0026	Wild-type	
**YOR369C**	RPS12	9.513	0.0026	Lower basal; no induction	N/A
**YLL026W**	HSP104	9.512	0.0026	Wild-type	
**YIL133C**	RPL16A	9.506	0.0055	Wild-type	
**YMR088C**	VBA1	9.505	0.0026		
**YDL042C**	SIR2	9.496	0.0045		Gfp⁻; no induction
**YNR075W**	COS10	9.490	0.0070	Wild-type	

**YIL025C**	YIL025C	9.370	0.0065	Wild-type	
**YBR032W**	YBR032W	9.283	0.0028	Lower basal	Wild-type
