# Supplementary material for: Barcode Sequencing Screen Identifies SUB1 as a Regulator of Yeast Pheromone Inducible Genes
Source: G3 (Bethesda). 2016 Feb 1;6(4):881–92. doi: 10.1534/g3.115.026757 (PMC4825658; doi:10.1534/g3.115.026757)
Supplement: Supporting Information [file supp_g3.115.026757_TableS3.pdf]

Table S3. Top genes enriched in "In Gfp^{basal}" population.
Genes in orange are expected to be enriched in this population; YJR087W* partially overlaps with *STE18*; shaded *kch1* and *whi3* mutants are a source of false positives and are described further in the text.

Systematic Name	Common Name	Log2 Fold Change	padj	Individually Tested Phenotype	Newly Made Mutant Phenotype
**YFL026W**	STE2	3.70176	1.06E-71		No induction
**YCL032W**	STE50	3.52653	1.61E-90		Lower basal; lower induced (See Figure 3)
**YJR087W***	YJR087W*	3.38461	2.78E-35		Lower basal; lower induced
**YNL197C**	WHI3	2.82035	6.48E-52	Very low basal; modest induction	Lower basal; lower induced
**YJL157C**	FAR1	2.72306	5.50E-40	Wild-type	
**YOR246C**	ENV9	2.28854	2.38E-31	Lower basal	Lower basal
**YHL007C**	STE20	2.22878	5.57E-21		Lower basal; lower induced (See Figure 3)
**YIL043C**	CBR1	2.05992	1.50E-34	Lower basal	Lower basal
**YIL116W**	HIS5	1.97992	3.66E-31	Wild-type	
**YJR054W**	KCH1	1.97460	1.15E-27	Lower basal; lower induced	Wild-type
**YDR410C**	STE14	1.95821	1.04E-58		Lower basal
**YOR144C**	ELG1	1.90017	6.74E-18		
**YIL055C**	YIL055C	1.89938	5.08E-50	Wild-type	
**YIL101C**	XBP1	1.84533	2.22E-33	Wild-type	
**YIL123W**	SIM1	1.83481	2.55E-10	Wild-type	
**YCL060C**	YCL060C	1.82481	2.66E-06		
**YDR181C**	SAS4	1.69610	1.83E-06		
**YMR052W**	FAR3	1.67344	3.96E-12	Wild-type	
**YIL160C**	POT1	1.64078	4.15E-15	Wild-type	
**YJL142C**	IRC9	1.63983	5.73E-07		
**YBL039C**	URA7	1.63740	7.54E-11		
**YDL021W**	GPM2	1.61446	1.54E-07	No induction	Wild-type
**YNL127W**	FAR11	1.60648	9.23E-07		
**YDR102C**	YDR102C	1.57243	2.46E-25		
**YIL111W**	COX5B	1.56441	1.51E-17	Wild-type	
**YHR108W**	GGA2	1.55728	2.92E-13		
**YMR029C**	FAR8	1.55177	1.21E-05	Wild-type	
**YOR083W**	WHI5	1.54981	3.60E-15	Wild-type	
**YGL032C**	AGA2	1.54217	1.21E-08	Wild-type	
