# Supplementary material for: Barcode Sequencing Screen Identifies SUB1 as a Regulator of Yeast Pheromone Inducible Genes
Source: G3 (Bethesda). 2016 Feb 1;6(4):881–92. doi: 10.1534/g3.115.026757 (PMC4825658; doi:10.1534/g3.115.026757)
Supplement: Supporting Information [file supp_g3.115.026757_TableS4.pdf]

Table S4. Top genes enriched in "Un Gfp+" population.
Shaded *sub1* mutant was identified in this study as having higher basal p*FUS1*-GFP reporter expression.

Systematic Name	Common Name	Log2 Fold Change	padj	Individually Tested Phenotype	Newly Made Mutant Phenotype
**YNL049C**	SFB2	5.652	1.38E-08	Wild-type	
**YDR391C**	YDR391C	5.497	3.24E-07		
**YOR378W**	YOR378W	5.311	1.38E-12	Wild-type	
**YDL197C**	ASF2	5.242	2.00E-06		
**YCL025C**	AGP1	5.130	2.28E-05		
**YOR342C**	YOR342C	5.109	1.88E-06	Wild-type	
**YBR090C**	YBR090C	4.936	3.75E-09	Wild-type	
**YNL299W**	TRF5	4.853	9.27E-06	No Induction	Wild-type
**YOR093C**	YOR093C	4.836	2.83E-07	Wild-type	
**YIL136W**	OM45	4.832	1.42E-06	Wild-type	
**YBL063W**	KIP1	4.721	3.54E-11	Wild-type	
**YJR019C**	TES1	4.496	1.74E-08	Lower basal	Wild-type
**YLL015W**	BPT1	4.331	7.07E-06	Wild-type	
**YNL271C**	BNI1	3.935	6.11E-61	Higher basal; higher induced	
**YDR192C**	NUP42	3.663	3.72E-18		
**YOR279C**	RFM1	3.559	3.77E-67		
**YLL047W**	YLL047W	3.521	6.14E-09	Wild-type	
**YPL248C**	GAL4	3.497	1.86E-07		
**YLR064W**	PER33	3.460	3.76E-09		
**YPR031W**	NTO1	3.458	9.32E-06		
**YLR093C**	NYV1	3.417	1.22E-05		
**YOL068C**	HST1	3.323	1.75E-06		
**YGL254W**	FZF1	3.321	4.49E-07	Wild-type	
**YER053C**	PIC2	3.238	1.62E-10		
**YPL246C**	RBD2	3.122	5.07E-08		
**YMR039C**	SUB1	3.122	0.0033	Higher basal	Higher basal
