# Supplementary material for: Barcode Sequencing Screen Identifies SUB1 as a Regulator of Yeast Pheromone Inducible Genes
Source: G3 (Bethesda). 2016 Feb 1;6(4):881–92. doi: 10.1534/g3.115.026757 (PMC4825658; doi:10.1534/g3.115.026757)
Supplement: Supporting Information [file supp_g3.115.026757_TableS5.pdf]

Table S5. Top genes enriched in "In Gfp++" population.
Shaded *sub1* mutant was identified in this study as having higher basal p*FUS1*-GFP reporter expression (data from a single experiment).

Systematic Name	Common Name	Log2 Fold Change	padj	Individually Tested Phenotype	Newly Made Mutant Phenotype
**YLL047W**	YLL047W	1.807	2.36E-44	Wild-type	
**YJR019C**	TES1	1.743	1.09E-26	Lower basal	Wild-type
**YOR378W**	YOR378W	1.462	1.42E-28	Wild-type	
**YGR206W**	MVB12	1.399	9.04E-12	Wild-type	
**YNL049C**	SFB2	1.389	8.84E-11	Wild-type	
**YER106W**	MAM1	1.333	9.98E-07	Wild-type	
**YMR039C**	SUB1	1.284	6.42E-05	Higher basal	Higher basal
**YBL063W**	KIP1	1.268	2.25E-21	Wild-type	
**YDR391C**	YDR391C	1.225	2.70E-12		
**YFL012W**	YFL012W	1.205	3.79E-06	Wild-type	
**YAL007C**	ERP2	1.201	2.86E-09	Wild-type	
**YBR016W**	YBR016W	1.200	7.45E-06		
**YMR026C**	PEX12	1.182	0.0333	Wild-type	
**YML081W**	TDA9	1.179	2.27E-05		
**YMR011W**	HXT2	1.178	0.0005	Wild-type	
**YAR047C**	YAR047C	1.174	1.18E-09	Wild-type	
**YLR018C**	POM34	1.170	0.0069	Wild-type	
**YML037C**	YML037C	1.168	2.49E-08	Wild-type	
**YIR028W**	DAL4	1.161	0.0008		
**YAL055W**	PEX22	1.159	1.21E-05	Wild-type	
**YML058C-A**	YML058C-A	1.156	1.19E-10	Wild-type	
**YNL146W**	YNL146W	1.152	4.24E-05	Gfp⁻; no induction	Wild-type
**YKL197C**	PEX1	1.144	1.73E-05		
**YNL305C**	BXI1	1.142	1.42E-05	Wild-type	
