# Supplementary material for: Barcode Sequencing Screen Identifies SUB1 as a Regulator of Yeast Pheromone Inducible Genes
Source: G3 (Bethesda). 2016 Feb 1;6(4):881–92. doi: 10.1534/g3.115.026757 (PMC4825658; doi:10.1534/g3.115.026757)
Supplement: Supporting Information [file supp_g3.115.026757_TableS6.pdf]

Table S6a. GFP fluorescence values of mating mutants in Figure 3.
These mutants were made by disrupting the indicated gene with *URA3*.

	Replicate 1	Replicate 2	Replicate 3	Mean	pvalue	Parent strain	Genotype
Negative Control BY4741, Un	3974	3955	3822	3917			*leu2Δ0 met15Δ0 ura3Δ0 his3Δ1*
Negative Control BY4741, In	3688	3603	4069	3786.667			*leu2Δ0 met15Δ0 ura3Δ0 his3Δ1*
Positive Control yAS38, Un	56109	51572	55000	54227			*leu2Δ0 met15Δ0 ura3Δ0 his3Δ1 bar1::pFus1-GFP-LEU2*
Positive Control yAS38, In	5.29E+05	6.89E+05	5.95E+05	6.04E+05			*leu2Δ0 met15Δ0 ura3Δ0 his3Δ1 bar1::pFus1-GFP-LEU2*
dig1, Un	63300	64245	61138	62894.333	0.00623	yAS38	*dig1Δ::URA3 leu2Δ0 met15Δ0 ura3Δ0 his3Δ1*
dig1, In	6.46E+05	6.39E+05	6.56E+05	647000	0.412	yAS38	*dig1Δ::URA3 leu2Δ0 met15Δ0 ura3Δ0 his3Δ1*
dig2, Un	41035	39913	43066	41338	0.00144	yAS38	*dig2Δ::URA3 leu2Δ0 met15Δ0 ura3Δ0 his3Δ1*
dig2, In	7.19E+05	7.09E+05	7.07E+05	711666.667	0.0825	yAS38	*dig2Δ::URA3 leu2Δ0 met15Δ0 ura3Δ0 his3Δ1*
fus3, Un	88301	88423	90833	89185.667	2.57E-05	yAS38	*fus3Δk::URA3 leu2Δ0 met15Δ0 ura3Δ0 his3Δ1*
fus3, In	4.44E+05	4.72E+05	4.20E+05	445333.333	0.0311	yAS38	*fus3Δk::URA3 leu2Δ0 met15Δ0 ura3Δ0 his3Δ1*
sir1, Un	16165	17262	20535	17987.333	4.40E-05	yAS38	*sir1Δ::URA3 leu2Δ0 met15Δ0 ura3Δ0 his3Δ1*
sir1, In	26394	38328	34506	33076	0.000253	yAS38	*sir1Δ::URA3 leu2Δ0 met15Δ0 ura3Δ0 his3Δ1*
sir2, Un	5420	5612	5315	5449	3.670E-06	yAS38	*sir2Δ::URA3*

							leu2Δ0 met15Δ0 ura3Δ0 his3Δ1
**sir2, In**	5377	5465	5354	5398.667	0.000208	yAS38	*sir2Δ::URA3 leu2Δ0 met15Δ0 ura3Δ0 his3Δ1*
**ste2, Un**	56435	56641	55791	56289	0.212	yAS38	*ste2Δ::URA3 leu2Δ0 met15Δ0 ura3Δ0 his3Δ1*
**ste2, In**	59623	61286	60690	60533	0.000304	yAS38	*ste2Δ::URA3 leu2Δ0 met15Δ0 ura3Δ0 his3Δ1*
**ste3, Un**	47584	49095	46215	47631.333	0.0146	yAS38	*ste3Δ::URA3 leu2Δ0 met15Δ0 ura3Δ0 his3Δ1*
**ste3, In**	6.97E+05	7.74E+05	7.96E+05	755666.667	0.0520	yAS38	*ste3Δ::URA3 leu2Δ0 met15Δ0 ura3Δ0 his3Δ1*
**ste4, Un**	61410	62183	58905	60832.667	0.0173	yAS38	*ste4Δ::URA3 leu2Δ0 met15Δ0 ura3Δ0 his3Δ1*
**ste4, In**	6.39E+05	6.07E+05	6.26E+05	624000	0.699	yAS38	*ste4Δ::URA3 leu2Δ0 met15Δ0 ura3Δ0 his3Δ1*
**ste5, Un**	5968	6136	6216	6106.667	3.89E-06	yAS38	*ste5Δ::URA3 leu2Δ0 met15Δ0 ura3Δ0 his3Δ1*
**ste5, In**	5740	5565	5633	5646	0.000208	yAS38	*ste5Δ::URA3 leu2Δ0 met15Δ0 ura3Δ0 his3Δ1*
**ste6, Un**	50461	50407	50085	50317.667	0.0463	yAS38	*ste6Δ::URA3 leu2Δ0 met15Δ0 ura3Δ0 his3Δ1*
**ste6, In**	7.42E+05	7.36E+05	7.54E+05	744000	0.0404	yAS38	*ste6Δ::URA3 leu2Δ0 met15Δ0 ura3Δ0 his3Δ1*
**ste13, Un**	43687	39854	38229	40590	0.00299	yAS38	*ste13Δ::URA3 leu2Δ0 met15Δ0 ura3Δ0 his3Δ1*
**ste13, In**	7.34E+05	7.11E+05	7.27E+05	724000	0.0633	yAS38	*ste13Δ::URA3 leu2Δ0 met15Δ0 ura3Δ0 his3Δ1*
**ste14, Un**	27590	29916	29729	29078.333	8.58E-05	yAS38	*ste14Δ::URA3 leu2Δ0 met15Δ0 ura3Δ0 his3Δ1*
**ste14, In**	6.69E+05	6.67E+05	6.56E+05	664000	0.270	yAS38	*ste14Δ::URA3*

				0			*leu2Δ0 met15Δ0 ura3Δ0 his3Δ1*
**ste20, Un**	11725	13814	13936	13158.333	1.18E-05	yAS38	*ste20Δ::URA3 leu2Δ0 met15Δ0 ura3Δ0 his3Δ1*
**ste20, In**	28175	35014	34960	32716.333	0.000251	yAS38	*ste20Δ::URA3 leu2Δ0 met15Δ0 ura3Δ0 his3Δ1*
**ste23, Un**	51048	46019	46675	47914	0.0390	yAS38	*ste23Δ::URA3 leu2Δ0 met15Δ0 ura3Δ0 his3Δ1*
**ste23, In**	7.47E+05	7.38E+05	7.30E+05	738333.333	0.0454	yAS38	*ste23Δ::URA3 leu2Δ0 met15Δ0 ura3Δ0 his3Δ1*
**ste24, Un**	34709	33392	32364	33488.333	0.000169	yAS38	*ste24Δ::URA3 leu2Δ0 met15Δ0 ura3Δ0 his3Δ1*
**ste24, In**	7.00E+05	6.97E+05	7.04E+05	700333.333	0.108	yAS38	*ste24Δ::URA3 leu2Δ0 met15Δ0 ura3Δ0 his3Δ1*

Table S6b. GFP fluorescence values of mating mutants in Figure 3, continued.

	Δ marker	Replicate 1	Replicate 2	Replicate 3	Mean	pvalue	Parent strain	Genotype
Negative Control BY4741, Un	*kanMX*	3952	4049	3976	3992.333		yAS38	*leu2Δ0 met15Δ0 ura3Δ0 his3Δ1*
Negative Control BY4741, In	*kanMX*	3984	3892	3868	3914.667		yAS38	*leu2Δ0 met15Δ0 ura3Δ0 his3Δ1*
Positive Control yAS38, Un	*kanMX*	57279	58776	55558	57204.333		yAS38	*leu2Δ0 met15Δ0 ura3Δ0 his3Δ1 bar1::pFus1-GFP-LEU2*
Positive Control yAS38, In	*kanMX*	4.81E+05	4.90E+05	4.85E+05	485333.333		yAS38	*leu2Δ0 met15Δ0 ura3Δ0 his3Δ1 bar1::pFus1-GFP-LEU2*
kss1, Un	*kanMX*	83433	84598	82443	83491.333	1.95E-05	yAS38	*kss1Δ::kanMX leu2Δ0 met15Δ0 ura3Δ0 his3Δ1*
kss1, In	*kanMX*	5.80E+05	5.92E+05	5.20E+05	564000	0.0247	yAS38	*kss1Δ::kanMX leu2Δ0 met15Δ0 ura3Δ0 his3Δ1*
ste4, Un	*kanMX*	5699	5246	5577	5507.333	6.53E-07	yAS38	*ste4Δ::kanMX leu2Δ0 met15Δ0 ura3Δ0 his3Δ1*
ste4, In	*kanMX*	5510	5154	5483	5382.333	5.21E-09	yAS38	*ste4Δ::kanMX leu2Δ0 met15Δ0 ura3Δ0 his3Δ1*
ste7, Un	*kanMX*	5135	5168	5168	5157	6.10E-07	yAS38	*ste7Δ::kanMX leu2Δ0 met15Δ0 ura3Δ0 his3Δ1*
ste7, In	*kanMX*	5051	4936	5029	5005.333	5.18E-09	yAS38	*ste7Δ::kanMX leu2Δ0 met15Δ0 ura3Δ0 his3Δ1*
ste8/sir3, Un	*URA3*	5151	5380	7667	6066	1.991E-06	yAS38	*steΔ8::kanMX leu2Δ0 lys2Δ0 ura3Δ0 his3Δ1 met15Δ0 bar1::pFUS1-GFP-LEU2*

**ste8/sir3, In**	*URA3*	4504	4656	6226	5128.667	5.66E-09	yAS38	*steΔ8::kanMX leu2Δ0 lys2Δ0 ura3Δ0 his3Δ1 met15Δ0 bar1::pFUS1-GFP-LEU2*
**ste9/sir4, Un**	*kanMX*	4655	4481	4359	4498.333	5.90E-07	yAS38	*ste9/sir4Δ::kanMX leu2Δ0 met15Δ0 ura3Δ0 his3Δ1*
**ste9/sir4, In**	*kanMX*	4451	4284	3988	4241	5.17E-09	yAS38	*ste9/sir4Δ::kanMX leu2Δ0 met15Δ0 ura3Δ0 his3Δ1*
**ste11, Un**	*kanMX*	5474	5264	5311	5349.667	6.24E-07	yAS38	*ste11Δ::kanMX leu2Δ0 met15Δ0 ura3Δ0 his3Δ1*
**ste11, In**	*kanMX*	5227	5196	5153	5192	5.19E-09	yAS38	*ste11Δ::kanMX leu2Δ0 met15Δ0 ura3Δ0 his3Δ1*
**ste12, Un**	*kanMX*	4085	3950	4058	4031	5.62E-07	yAS340	*ste12Δ::kanMX leu2Δ0 lys2Δ0 ura3Δ0 his3Δ1 bar1::pFUS1-GFP-LEU2*
**ste12, In**	*kanMX*	3733	3673	3697	3701	5.12E-09	yAS340	*ste12Δ::kanMX leu2Δ0 lys2Δ0 ura3Δ0 his3Δ1 bar1::pFUS1-GFP-LEU2*
**ste18, Un**	*kanMX*	5885	5936	5603	5808	6.57E-07	yAS340	Replicate 1: *ste18Δ::kanMX leu2Δ0 ura3Δ0 his3Δ1 met15Δ0 bar1::pFUS1-GFP-LEU2*<br><br>Replicates 2 and 3: *ste18Δ::kanMX leu2Δ0 lys2Δ0 ura3Δ0 his3Δ1 met15Δ0 bar1::pFUS1-GFP-LEU2*
**ste18, In**	*kanMX*	5365	5479	5262	5368.667	5.199E-09	yAS340	Replicate 1: *ste18Δ::kanMX leu2Δ0 ura3Δ0*

								his3Δ1 met15Δ0 bar1::pFUS1-GFP-LEU2<br><br>Replicates 2 and 3: *ste18Δ::kanMX leu2Δ0 lys2Δ0 ura3Δ0 his3Δ1 met15Δ0 bar1::pFUS1-GFP-LEU2*
**ste21, Un**	*kanMX*	18732	20256	19327	19438.333	3.304E-06	yAS38	*ste21Δ::kanMX leu2Δ0 met15Δ0 ura3Δ0 his3Δ1*
**ste21, In**	*kanMX*	204612	201627	235408	213882.333	1.663E-05	yAS38	*ste21Δ::kanMX leu2Δ0 met15Δ0 ura3Δ0 his3Δ1*
**ste22, Un**	*kanMX*	47794	50171	51062	49675.667	0.005	yAS38	*ste22Δ::kanMX leu2Δ0 met15Δ0 ura3Δ0 his3Δ1*
**ste22, In**	*kanMX*	4.79E+05	4.84E+05	4.95E+05	486000	0.172	yAS38	*ste22Δ::kanMX leu2Δ0 met15Δ0 ura3Δ0 his3Δ1*
**ste50, Un**	*kanMX*	9754	10005	11321	10360	1.504E-06	yAS38	*ste50Δ::kanMX leu2Δ0 met15Δ0 ura3Δ0 his3Δ1*
**ste50, In**	*kanMX*	37826	40050	41722	39866	9.874E-09	yAS38	*ste50Δ::kanMX leu2Δ0 met15Δ0 ura3Δ0 his3Δ1*
