# Supplementary material for: Barcode Sequencing Screen Identifies SUB1 as a Regulator of Yeast Pheromone Inducible Genes
Source: G3 (Bethesda). 2016 Feb 1;6(4):881–92. doi: 10.1534/g3.115.026757 (PMC4825658; doi:10.1534/g3.115.026757)
Supplement: Supporting Information [file supp_g3.115.026757_TableS7.pdf]

Table S7: Strains used in this study.

Description	Strain	Genotype	Figure
**Negative control strain**	**BY4741**	*leu2Δ0 met15Δ0 ura3Δ0 his3Δ1*	
**Reporter control strain**	**yAS38**	*leu2Δ0 met15Δ0 ura3Δ0 his3Δ1 bar1::pFus1-GFP-LEU2*	
**BY4743 transformed with p*FUS1*-GFP reporter construct**	**yAS340**	*leu2Δ0 lys2Δ0/+ ura3Δ0 his3Δ1 met15Δ0/+ bar1::pFUS1-GFP-LEU2*	
**SUB1–3HA tag for ChIP-Seq**	**yAS420**	*SUB1-3HAtag::kanMX leu2Δ0 lys2Δ0 ura3Δ0 his3Δ1 met15 bar1::pFUS1-GFP-LEU2*	
***sub1Δ*::kanMX for RNA-Seq**	**yAS395**	*sub1Δ::kanMX lys2Δ0 ura3Δ0 his3Δ1 met15 bar1::pFUS1-GFP-LEU2*	
**WT control for RNA-Seq**	**yAS418**	*lys2Δ0 ura3Δ0 his3Δ1 met15 bar1::pFUS1-GFP-LEU2*	
	**yAS473**	*hog1Δ::hygMX leu2Δ0 ura3Δ0 his3Δ1 bar1::pFus1-GFP-LEU2*	**Figure 4**
	**yAS474**	*sub1Δ::kanMX hog1Δ::hygMX leu2Δ0 ura3Δ0 his3Δ1 met15 bar1::pFus1-GFP-LEU2*	**Figure 4**
	**yAS476**	*sub1Δ::kanMX leu2Δ0 lys2Δ0 ura3Δ0 his3Δ1 bar1::pFus1-GFP-LEU2*	**Figure 4**
whi3Δ strain from *MAT*a YKO library	**yAS146**	*whi3Δ::kanMX leu2Δ0 met15Δ0 ura3Δ0 his3Δ1 bar1::pFus1-GFP-LEU2*	**Figure S6**
whi3Δ strain from *MAT*a YKO library transformed with reporter	**yAS170**	*whi3Δ::kanMX leu2Δ0 met15Δ0 ura3Δ0 his3Δ1 bar1::pFus1-GFP-LEU2*	**Figure S6**
kch1Δ strain from *MAT*a YKO library	**yAS199**	*kch1Δ::kanMX leu2Δ0 met15Δ0 ura3Δ0 his3Δ1 bar1::pFus1-GFP-LEU2*	**Figure S6**
kch1Δ strain from *MAT*a YKO library	**yAS210**	*kch1Δ::kanMX leu2Δ0 met15Δ0 ura3Δ0 his3Δ1*	**Figure S6**

transformed with reporter		*bar1::pFus1-GFP-LEU2*	
	**yAS408**	*pbs2Δ::URA3 sub1Δ::kanMX leu2Δ0 lys2Δ0 ura3Δ0 his3Δ1*	**Figure S5**
	**yAS409**	*sub1Δ::kanMX leu2Δ0 ura3Δ0 his3Δ1 met15Δ0*	**Figure S5**
	**yAS410**	*pbs2Δ::URA3 leu2Δ0 lys2Δ0 ura3Δ0 his3Δ1*	**Figure S5**
	**yAS415**	*leu2Δ0 lys2Δ0 ura3Δ0 his3Δ1*	**Figure S5**
	**yAS454**	*pbs2Δ::URA3 SUB1-3HAtag::kanMX leu2Δ0 lys2Δ0 ura3Δ0 his3Δ1 met15Δ0*	**Figure S5**
