# Supplementary material for: Barcode Sequencing Screen Identifies SUB1 as a Regulator of Yeast Pheromone Inducible Genes
Source: G3 (Bethesda). 2016 Feb 1;6(4):881–92. doi: 10.1534/g3.115.026757 (PMC4825658; doi:10.1534/g3.115.026757)
Supplement: Supporting Information [file supp_g3.115.026757_FigureS2.pdf]

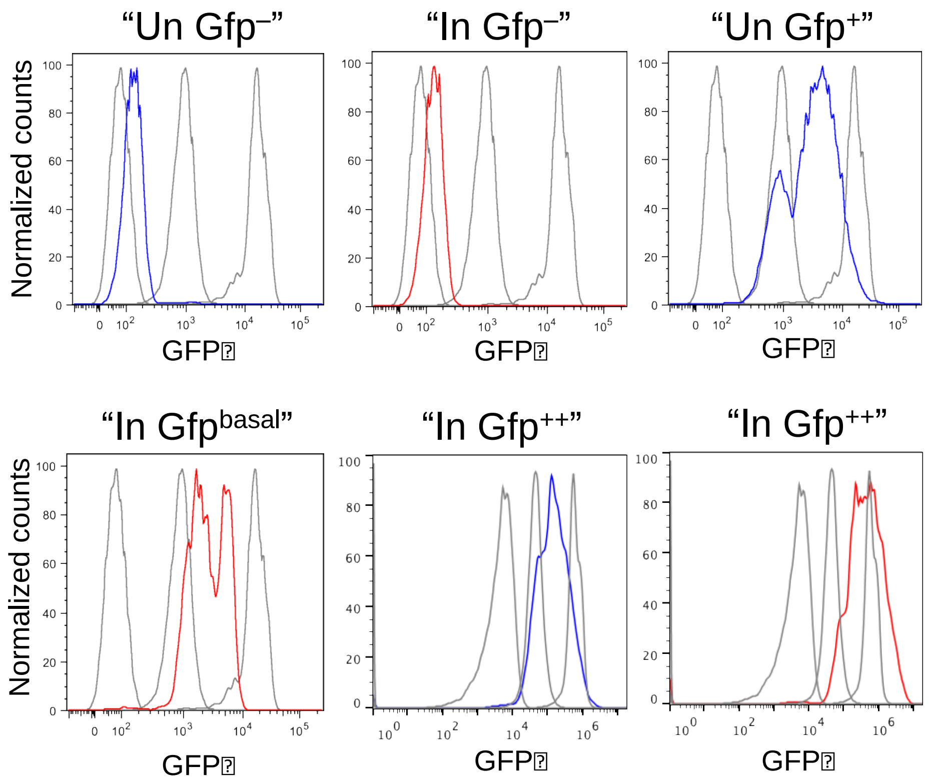

Figure S2 FACS analysis of populations after sorting.
Top Panel: FACS analysis of “Un Gfp–” population regrown in uninduced conditions after sorting; FACS analysis of “In Gfp–” population regrown in induced conditions after sorting; and FACS analysis of “Un Gfp+” population regrown in uninduced conditions after sorting.
Bottom Panel: FACS analysis of “In Gfp basal” population regrown in induced conditions after sorting; FACS analysis of “In Gfp++” population regrown in uninduced conditions; and FACS analysis of “In Gfp++” population regrown in induced conditions.
