# Supplementary material for: Barcode Sequencing Screen Identifies SUB1 as a Regulator of Yeast Pheromone Inducible Genes
Source: G3 (Bethesda). 2016 Feb 1;6(4):881–92. doi: 10.1534/g3.115.026757 (PMC4825658; doi:10.1534/g3.115.026757)
Supplement: Supporting Information [file supp_g3.115.026757_FigureS3.pdf]

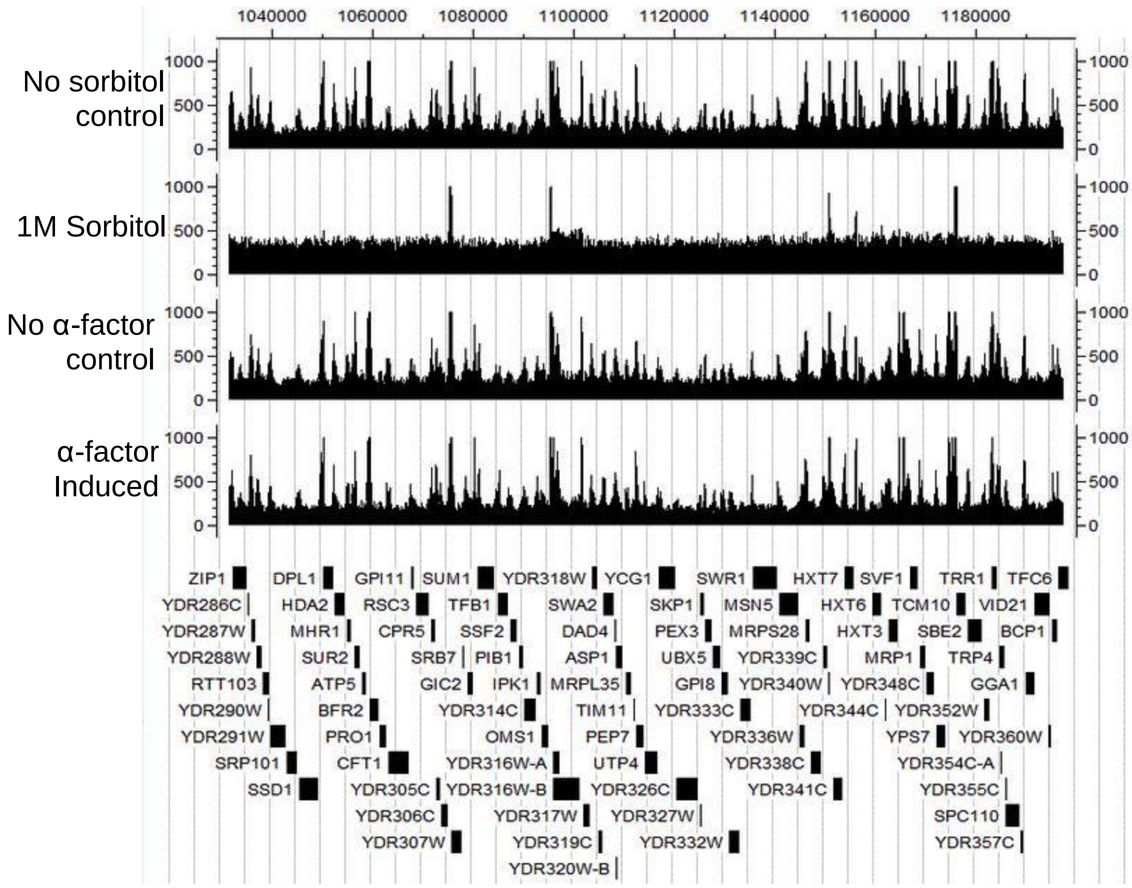

Figure S3 Screenshot of Sub1-3HA ChIP-Seq under high osmolarity conditions. ChIP-Seq of Sub1 shows that, overall, Sub1 releases binding genome-wide under high sorbitol conditions.
