# Supplementary figures and images for: Barcode Sequencing Screen Identifies SUB1 as a Regulator of Yeast Pheromone Inducible Genes
Source: G3 (Bethesda). 2016 Feb 1;6(4):881–92. doi: 10.1534/g3.115.026757 (PMC4825658; doi:10.1534/g3.115.026757)

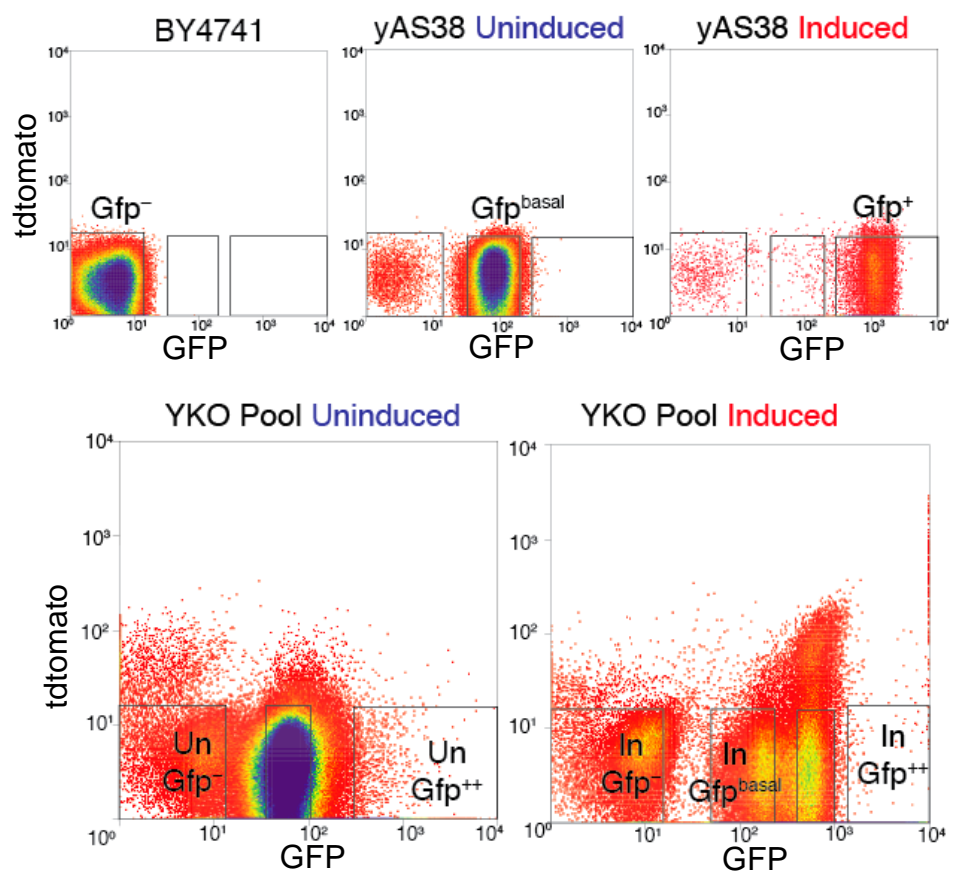

**Figure S4** Gating of YKO library sorting experiments.

Supplement: Supporting Information [file supp_g3.115.026757_FigureS4.pdf]

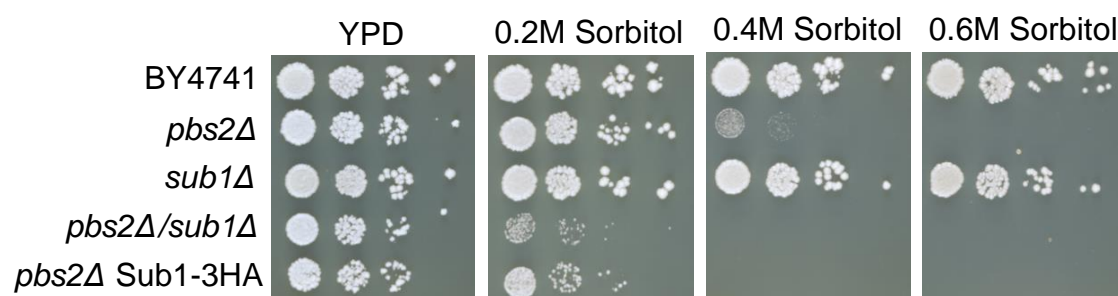

**Figure S5** SUB1-3HA tagged strain retains some Sub1 function.

Supplement: Supporting Information [file supp_g3.115.026757_FigureS5.pdf]
