# Supplementary material for: Barcode Sequencing Screen Identifies SUB1 as a Regulator of Yeast Pheromone Inducible Genes
Source: G3 (Bethesda). 2016 Feb 1;6(4):881–92. doi: 10.1534/g3.115.026757 (PMC4825658; doi:10.1534/g3.115.026757)
Supplement: Supporting Information [file supp_g3.115.026757_FigureS6.pdf]

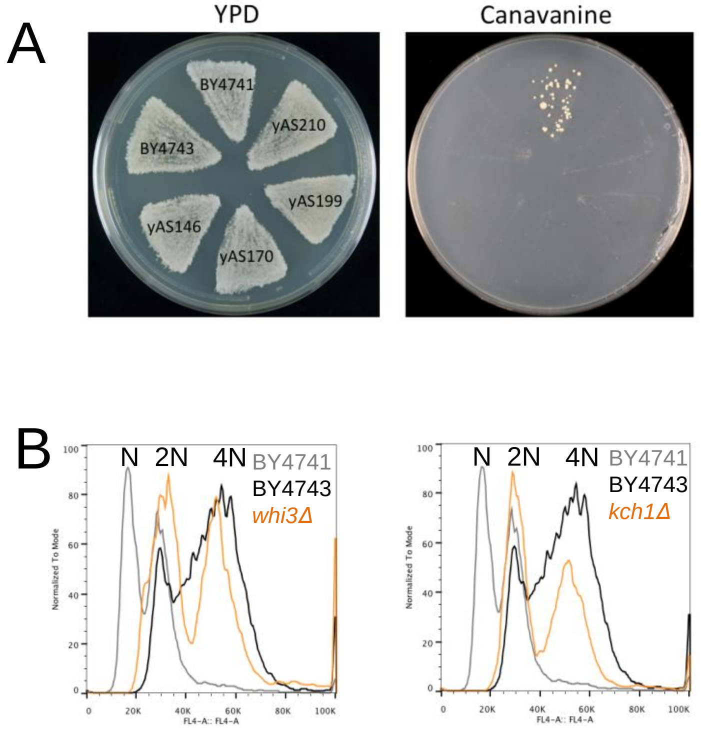

Figure S6 *whi3Δ* and *kch1Δ* mutants from the *MAT*a YKO library are diploid. (A) Testing for ploidy using the canavanine resistance test. Since canavanine resistance in yeast is conferred by a recessive mutation in the *CAN1* gene, haploid strains form resistant papillae on canavanine plates at a higher rate than diploid strains. Here, a haploid control (BY4741) forms canavanine resistant papillae, while a diploid control (BY4743) does not. The *whi3Δ* (yAS146 and yAS170) and *kch1Δ* (yAS199 and yAS210) strains from the *MAT***a** YKO library also do not form canavanine resistant papillae, consistent with the phenotype of a diploid strain. (B) Propidium iodide staining of the *whi3Δ* and *kch1Δ* strains from the YKO library shows that these strains have DNA content consistent with that of a diploid strain.
