# Supplementary material for: Barcode Sequencing Screen Identifies SUB1 as a Regulator of Yeast Pheromone Inducible Genes
Source: G3 (Bethesda). 2016 Feb 1;6(4):881–92. doi: 10.1534/g3.115.026757 (PMC4825658; doi:10.1534/g3.115.026757)
Supplement: Supporting Information [file supp_g3.115.026757_FigureS7.pdf]

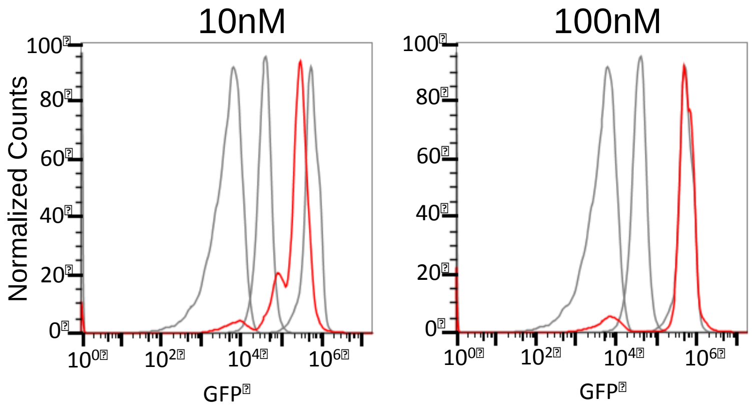


**Figure S7** Fluorescence peak of YKO library shifts with a higher concentration of α-factor. Adding more α-factor to the YKO library shifts peak of α-factor induced to wild-type levels (10nM vs. 100nM).
