# Supplementary material for: Barcode Sequencing Screen Identifies SUB1 as a Regulator of Yeast Pheromone Inducible Genes
Source: G3 (Bethesda). 2016 Feb 1;6(4):881–92. doi: 10.1534/g3.115.026757 (PMC4825658; doi:10.1534/g3.115.026757)
Supplement: Supporting Information [file supp_g3.115.026757_FigureS8.pdf]

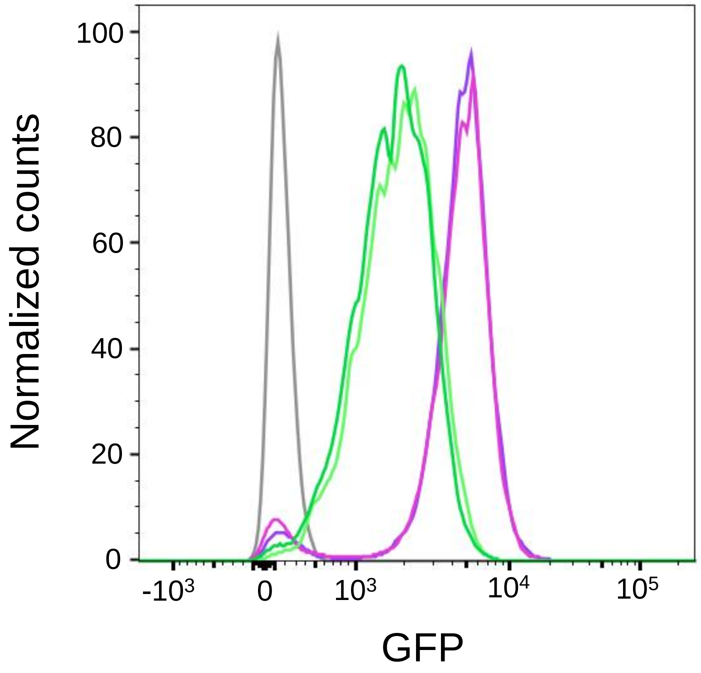

Strain	Mean GFP Fluorescence
yAS476 + Sub1 replicate 1	1894
yAS476 + Sub1 replicate 2	2061
yAS476 empty vector replicate 1	4827
yAS476 empty vector replicate 2	4950
BY4741 Negative Control	165

Figure S8 Plasmid rescue of *sub1Δ* mutant. This plasmid rescue experiment shows that wild-type expression of Sub1 from a pRS416 plasmid decreases the p*FUS1*-GFP expression in a *sub1Δ* mutant.
