# Supplementary material for: Barcode Sequencing Screen Identifies SUB1 as a Regulator of Yeast Pheromone Inducible Genes
Source: G3 (Bethesda). 2016 Feb 1;6(4):881–92. doi: 10.1534/g3.115.026757 (PMC4825658; doi:10.1534/g3.115.026757)
Supplement: Supporting Information [file supp_g3.115.026757_FigureS9.pdf]

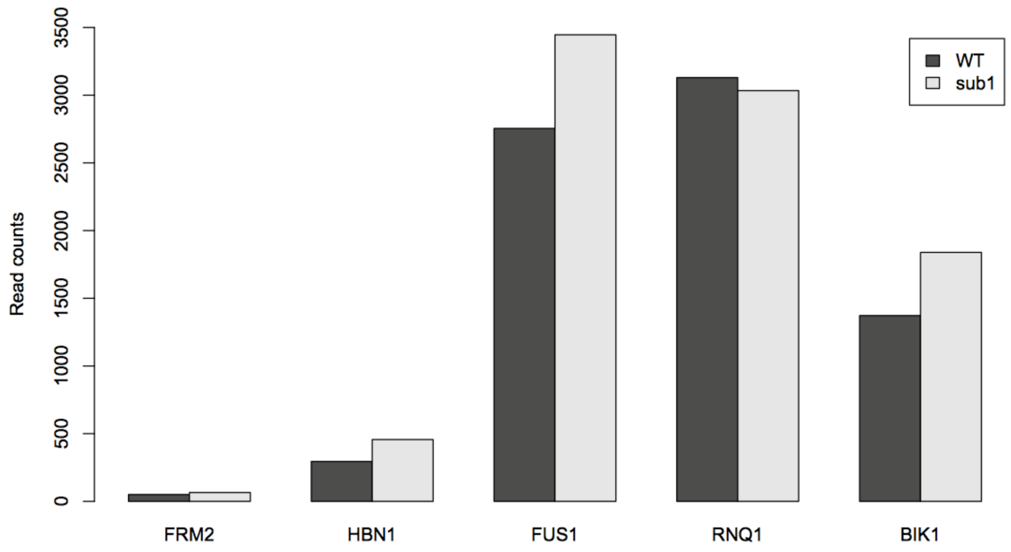

Figure S9 *FUS1* expression is higher in a *sub1Δ* mutant relative to wild-type. Additional computational analysis of RNA-Seq read counts shows that *FUS1* expression in a *sub1Δ* mutant is ~1.2 fold higher than wild-type.
